# Seasonal Reproduction in Vertebrates: Melatonin Synthesis, Binding, and Functionality Using Tinbergen’s Four Questions

**DOI:** 10.3390/molecules23030652

**Published:** 2018-03-13

**Authors:** Dax viviD, George E. Bentley

**Affiliations:** Berkeley Department of Integrative Biology, University of California, Berkeley, CA 94720, USA; gb7@berkeley.edu

**Keywords:** melatonin, seasonal reproduction, vertebrates

## Abstract

One of the many functions of melatonin in vertebrates is seasonal reproductive timing. Longer nights in winter correspond to an extended duration of melatonin secretion. The purpose of this review is to discuss melatonin synthesis, receptor subtypes, and function in the context of seasonality across vertebrates. We conclude with Tinbergen’s Four Questions to create a comparative framework for future melatonin research in the context of seasonal reproduction.

## 1. Introduction

“*We begin in the dark, and birth is the death of us.*” Antigonḗ, Translated by Anne Carson

The connection between darkness and birth is inextricable for photoperiodic, seasonally breeding animals. The tilt and rotation of the Earth creates geographical variation in temperature and daylength. Daily and annual fluctuations of environmental conditions outside of the tropics, resulting from these astronomical circumstances, correspond to changes in relative resource abundance. Organisms that detect light with the physiological mechanisms to track and store photic information over time can use daylength to anticipate environmental changes, subsequently altering behavior and physiology to optimize metabolism and seasonal reproductive timing. The synthesis of *N*-acetyl-5-methoxytryptamine, or melatonin, corresponds with darkness, thereby transducing photic information at the physiological level.

The focus of this review is to overview melatonin research in the context of seasonal reproduction in photoperiodic breeding mammals, birds, and other vertebrates. Melatonin synthesis, binding, and signalling in the hypothalamo-pituitary gonadal (HPG) axis is discussed within existing models for molecular regulation of seasonal reproduction. We conclude by using the framework set forth by Tinbergen (1963) for ethology to pose questions to guide future experiments studying the connection between melatonin and seasonal reproduction.

## 2. Melatonin Synthesis

Melatonin synthesis originated in mitochondria and chloroplasts [1]. The antioxidant cascade of byproducts in the biosynthetic pathway are speculated to serve a key role in evolutionary history. Derived from the amino acid tryptophan, melatonin is synthesized with four key enzymes. Enzymes in the biosynthetic pathway optimized functionality by peaking expression during the time of day with lower temperatures and UV radiation: nighttime. The main source of circulating melatonin in vertebrates is the pineal, and photic information, transmitted via the phototransduction pathway, is the primary regulator of pineal melatonin synthesis and secretion [2].

### 2.1. Phototransduction Pathways in Mammals and Birds

**Figure 1:**
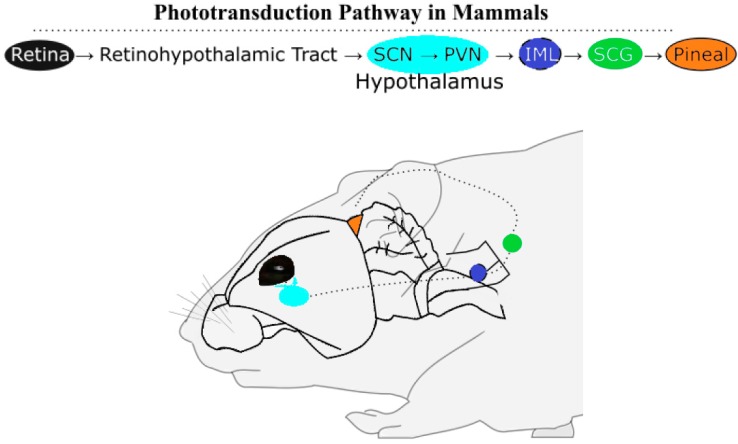
Phototransduction pathway in mammals. Light is detected by the retina. Photic information is transduced via the retinohypothalamic tract to nuclei in the hypothalamus. The suprachiasmatic nucleus (SCN) projects to the paraventricular nucleus (PVN), which synapses in the superior cervical ganglion (SCG). The signal is transmitted from the SCG to the Pineal.

Melatonin is an established chemical transducer of photic information because its synthesis in photoreceptive organisms reaches its zenith in darkness. The inverse relationship between day length and the length of the subjective night, which varies depending on the season outside of the tropics, is translated through the duration of melatonin synthesis at night. The duration of melatonin synthesis drives the reproductive state in a number of seasonal, photoperiodic breeding mammals. Among these mammals, there are short-day breeders that breed in winter (e.g., sheep) and long-day breeders that breed in summer (e.g., hamsters). The gestation lengths vary to enable parturition at the predicted time of year with highest resource abundance, springtime. Long winter nights, corresponding to an extended duration of melatonin synthesis, stimulate the reproductive axis of short-day breeders and inhibit long-day breeders. In hamsters, a long-day breeder, induced testicular regression observed in extended darkness can be prevented by removing the pineal gland or through bilateral enucleation, illustrating the role light plays in reproductive state [3]. Pinealectomies remove a significant portion of circulating melatonin across vertebrates [4], and the pineal gland continues to be researched as a key component of the pathway (see Figure 1) for photic and endogenous regulation of mammalian melatonin synthesis [5,6].

Even in the wild, the impacts of artificial light at night suppresses melatonin levels and affect reproductive physiology in the tammer wallaby (*Macropus eugenii*) [7] and European starlings (*Sturnus vulgaris*) [8], indicating there are ecological implications of the effects of light and melatonin on mammalian and avian breeding cycles. However, phototransduction in birds includes pineal and deep-brain photoreceptors situated beneath a translucent skull, whereas in mammals photic information is transduced via the retino-hypothalamic pathway. Benoît conducted several experiments in ducks (*Anas platyrhynchos*) demonstrating that deep brain photoreception was sufficient to induce testicular development if the light administered includes blue wavelengths [9,10]. Bilateral enucleation and pinealectomy in tree sparrows (*Spizella arborea*) did not prevent testicular growth on long days [11]. Although this study removes the main sources of circulating melatonin, detectable levels of melatonin has been measured in plasma after removal of the eyes and pineal in quail (*Coturnix japonica*). One-third of quail without these photoreceptive organs could still entrain to a light-dark cycle [12]. These findings imply that melatonin synthesis occurs in yet another photoreceptive site in birds. Given that the other photoreceptive sites of birds, the eyes and pineal, use melatonin to transduce photic information, deep brain photoreceptors of the hypothalamus use the same chemical signal. In fact, turkey (*Meleagris gallopavo*) have melanopsin photoreceptors in the premammillary nucleus of the hypothalamus, along with key melatonin-synthesizing enzymes [13,14] Deep-brain photoreceptors in chicken (*Gallus gallus*) are capable of driving gonadal response [15], in line with findings observed nearly fifty years ago in ducks [10]. The molecular mechanism of how light regulates melatonin synthesis can be understood through regulation of melatonin-synthesizing enzymes.

### 2.2. Melatonin-Synthesizing Enzymes

**Figure 2:**
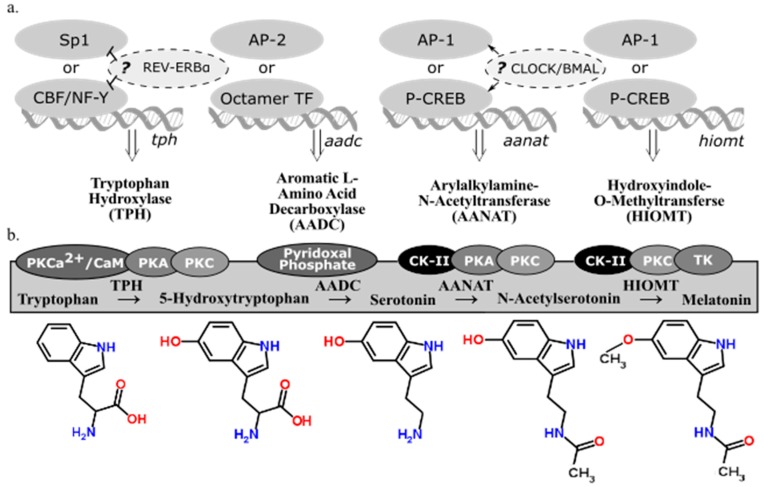
Transcription (**a**) and activation (**b**) of melatonin-synthesizing enzymes. Transcription factors of *tph* include Sp1 and CBF/NF-Y complex [17]. REV-ERBα inhibits *tph* transcription [18], implying some connection with circadian regulation, but it is not known through what mechanism. TPH is phosphorylated/activated by calmodulin (CaM), phosphokinase A (PKA) and phosphokinase C (PKC) [17]. Transcription factors of *aadc* can include AP-2 or octamer transcription factors [19], neither of which are known for circadian regulation. AADC depends on pyridoxal phosphate for functionality [20]. Transcription factors of *aanat* and *hiomt* include AP-1 and phosphorylated CREB (P-CREB). The *aanat* gene is likely regulated in a circadian fashion via the CLOCK/BMAL heterodimer [17]. AANAT and HIOMT have binding sites for casein kinase type II (CK-II) and PKC, and activation of HIOMT is enabled by tyrosine kinase (TK) [17].

There are four enzymes involved in melatonin synthesis from its amino acid precursor, tryptophan: tryptophan hydroxylase, dopa-decarboxylase, arylalkylamine-*N*-acetyltransferase, and hydroxyindole-*O*-methyltransferase (see summary in Figure 2). The nomenclature of these enzymes and their variants, isozymes with the same functional roles but differentially regulated, continue to be modified as we learn more about when and how they are transcribed and translated. This overview accounts for how melatonin-synthesizing enzymes are regulated at the the levels of transcription (Figure 2a) and activation (Figure 2b). A more detailed account of post-transcriptional modifications of these genes can be found in a book compiled by Pandi-Perumal and Cardinali [16].

### 2.3. Tryptophan Hydroxylase

Tryptophan hydroxylase (TPH) initiates the melatonin biosynthetic pathway, and it is regarded as the rate-limiting enzyme in serotonin (5-HT) synthesis [21]. The CCAAT box binding factor (CBF)/NF-Y complex is involved in transcriptional activation of *tph* in human DNA derived from P815-HTR and HeLa nuclear protein extracts [22]. Because NF-Y contributes to disease-response and photoreceptor cell differentiation in *Drosophila* [23], the possibility of TPH regulation in congruence with these processes is plausible. Specialty protein 1 (Sp1) is another known transcription factor for the *tph* gene [17]. In *Xenopus laevis*, *tph* expression in the retina is considered to be under circadian control because its expression fluctuates in a circadian fashion even in constant darkness [24]. Additionally, transcriptional suppression of *tph* and the clock gene *bmal1* is mediated through circadian nuclear receptor REV-ERBα, shown through significant differences of *tph* expression observed in wild type and *Rev-erbα* KO mice [18]. Given the functional similarities of TPH variants, TPH1 (NCBI Gene ID: 7166) and TPH2 (NCBI Gene ID: 121278), little has been done to distinguish transcriptional regulation of these two genes, found on different chromosomes in humans. Given that TPH plays a role in both serotonin and melatonin synthesis from the precursor, tryptophan, the end product to which transcriptional regulation of *tph* is directed cannot be determined. Therefore, transcriptional regulation of subsequent enzymes in the melatonin biosynthetic pathway must be considered alongside TPH.

### 2.4. Aromatic l-Amino Acid Decarboxylate

The second enzyme in the melatonin biosynthetic pathway, dopa decarboxylase (DDC; NCBI Gene ID: 1644), or aromatic l-amino acid decarboxylate (AADC), decarboxylates the product of TPH (5-hydroxytryptophan) to synthesize 5-hydroxytryptamine (5-HT), serotonin. *Aadc* gene regulation varies depending on the tissue considered [25]. The promoter regions of *ddc* has putative binding sites for octamer transcription factors (TF) and AP-2, suggesting alternative regulatory pathways [19]; however, neither of these transcription factors are associated with circadian regulation. AADC depends on pyridoxal phosphate for functionality [20]. Because AADC is the rate-limiting step in neither serotonin nor melatonin synthesis, research on its regulatory mechanisms is sparse compared to the amount of research on the transcriptional regulation of the penultimate enzyme in the melatonin biosynthetic pathway, AANAT.

### 2.5. Arylalkylamine-N-Acetyltransferase

Arylalkylamine-*N*-acetyltransferase (AANAT) or 5-HT-*N*-acetyltransferase (NCBI Gene ID: 15), is highly localized in the pineal gland [26] and converts serotonin into *N*-acetylserotonin (NAS). Evidence has been cited claiming that NAS is an antioxidant [27] with its own circadian rhythm that binds and activates the TrkB receptor [28]. Rhythmic transcriptional regulation of *Aanat* includes the cAMP response element modulator and its product, inducible cAMP early repressor (ICER) [29]. In rat pinealocytes, adrenergic-cAMP regulation upregulated pineal *Aanat* synthesis in darkness and inhibit its own synthesis during the light period [30]. Structure, function, and regulation in AANAT activity have been extensively reviewed [31] as well as *Aanat* transcriptional regulation via norepinephrine in the rat pineal gland [32]. The circadian expression of *Aanat* is significantly correlated to the expression of a rhythmic transcription factor of the cone-rod homeobox (Crx) gene, as seen from a study that overexpressed Crx, used adenovirus-mediated short hairpin RNA gene to knockdown Crx, and tested Crx-knockout mice and found a significant corresponding downregulation of *Aanat* expression [33]. There are detectable levels of *Aanat* expression in the retina [34] and the rat brain [30]; however, the exact neural regions were not isolated in these particular studies. If comparable transcriptional regulatory mechanisms apply to other sites of *Aanat* synthesis, then *Aanat* expression can be regulated by light along the phototransduction pathway. This pathway includes specific nuclei of the hypothalamus in some mammals (e.g., the suprachiasmatic nucleus, or SCN, and paraventricular nucleus, or PVN) or deep-brain photoreceptors in birds (e.g., the premammillary nucleus). Interestingly, rhythmic melatonin synthesis in the sheep pineal did not correlate to *Aanat* gene expression, suggesting that sheep regulate pineal melatonin synthesis in a manner that differs from rodents or long-day breeders in general [35]. The AANAT protein has binding sites for casein kinase type II (CK-II), PKA, and PKC [17]. Since CK-II phosphorylates PER2 in a circadian fashion [36], CK-II refines circadian regulation of AANAT phosphorylation. An investigation into the evolution of AANAT revealed different subtypes across vertebrates [37,38,39].

### 2.6. Hydroxyindole-O-Methyltransferase

The final enzyme of the melatonin biosynthetic pathway, hydroxyindole-*O*-methyltransferase (HIOMT, NCBI Gene ID: 438), synthesizes melatonin from NAS. Both light and time of day can influence *Hiomt* expression in the chicken pineal gland [40]. The avian pineal is directly photoreceptive while the mammalian pineal gland receives photic input via the phototransduction pathway. A radioenzymatic assay detected direct β_1_-adrenergic regulation of the *Hiomt* gene [40]. Additionally, AANAT might not be the rate-limiting enzyme in melatonin synthesis in rats, implying that HIOMT can play this role instead [41]. Although AANAT is still regarded as the enzyme that drives the circadian rhythm of melatonin synthesis in some contexts, the amplitude of nocturnal melatonin synthesis that fluctuates with annual photoperiod can be regulated by HIOMT. In Siberian hamsters housed in short photoperiods, HIOMT activity is significantly higher than hamsters housed in long photoperiods, so there might be a seasonal relationship between HIOMT activity and melatonin synthesis [42]. The HIOMT protein, like AANAT, has binding sites for CK-II, PKA, and PKC [17]. Because both enzymes are post-translationally activated by similar factors, it is possible that AANAT and HIOMT are both differentially regulated rate-limiting enzymes in the melatonin biosynthetic pathway.

### 2.7. Summary

The regulatory process of melatonin synthesis includes photoinhibition of photoreceptive sites as well as transcription, translation, and activation of melatonin-synthesizing enzymes. Instantaneous measurements of any one of these factors can differ based on the time of day and season of the year. The modes of transcription, translation, and activation reviewed here are not comparable across tissues nor conserved across species. To date, the melatonin biosynthetic pathway and the enzymes it comprises have not been studied enough to claim there is circadian regulation of enzyme expression across vertebrates.

The main trend of research on the molecular underpinnings of melatonin synthesis focuses on mammalian pineal melatonin synthesis. In the following sections of this review, extra-pineal sites that are capable of local synthesis and secretion of melatonin at undetectable levels in plasma are reviewed. This research elucidates novel supplements to pineal melatonin synthesis in seasonally breeding vertebrates. However, the endocrinological relevance of localized melatonin synthesis in extra-pineal tissue requires binding of melatonin and activation of its receptor.

## 3. Melatonin Binding

Once melatonin is synthesized, and its amphiphilic properties allow it to diffuse across the cellular membrane, it can either work as an antioxidant whose byproducts are a part of a cascade of free-radical scavengers [43] or as a hormone and bind specific receptors. Two approaches are commonly used to research the endocrine role of melatonin: (1) autoradiography radiolabeled ligands localize melatonin binding-sites or provide pharmacological evidence of melatonin receptor binding properties and (2) RNA extraction and/or in situ hybridization sequence and label mRNA coding for specific melatonin receptor subtypes. This section reviews recent findings from studies using these approaches to better understand melatonin binding. Additionally, the most extensive multiple sequence alignment of melatonin subtype receptors to date was conducted fill gaps in our comparative understanding of melatonin subtype receptors across vertebrates.

### 3.1. Melatonin and Autoradiography

Radioligand studies elucidate the prominence of melatonin binding in peripheral tissues in rodents [44] and in the brain across vertebrates [45,46,47]. The pars tuberalis of the anterior pituitary, as a conserved melatonin binding site in mammals, is a key site for understanding the phylogeny of seasonal timing [48]. Although the PT is a conserved site for melatonin-binding, the localization of membrane receptors for melatonin in the hypothalamus varies across species, reproductive states, and lighting conditions. In ferrets (*Mustela putorius furo*), melatonin only binds in the pituitary and not in the brain [49]. In other mammals, melatonin also binds in the hypothalamus. In Sprague-Dawley rats, the suprachiasmatic nucleus (SCN) of the hypothalamus and the median eminence (ME) have relatively higher melatonin radioligand binding [50]. Furthermore, in male Wistar rats, there were no differences in the density and affinity of melatonin binding in the PT and the SCN, and daily fluctuations in circulating melatonin levels can regulate melatonin receptors in these sites [51]. Melatonin binds the SCN and ME in addition to the preoptic area (POA) and dorsomedial region of ventromedial nuclei (VMN) of Syrian hamsters [52]. Even within the SCN, Syrian and Siberian hamsters show 2-[^125^I]iodomelatonin binding within regions that are not directly associated with the phototransduction pathway [53]. In C3H/HeN mice, 2-[^125^I]iodomelatonin binding in the SCN is significantly higher at 2 h after lights on during the subjective day [54], showing that the lighting condition, not independent of endogenously regulated circadian rhythms, affects melatonin binding in a given neural site. A non-rodent mammal commonly used to study melatonin’s role in seasonal reproductive timing is sheep (*Ovis aries*). An area with high melatonin binding in the sheep hypothalamus is the premammilary nucleus (PMM) [55]. Ablation and replacement, or lesions, of sites with high density of melatonin binding can provide information about the functional role of melatonin binding in a given site. However, lesions disrupt other aspects of the network unrelated to melatonin binding.

In non-mammalian vertebrates, daylength and reproductive state affect melatonin binding as well. Melatonin binding in the forebrain of European starlings is observed in nuclei associated with the song control system, such as Area X. Total 2-[^125^I]iodomelatonin binding was associated with reproductive state in starlings housed in the laboratory, not the lighting condition [56]. Annual fluctuations in the volume of these nuclei are affected by exogenous melatonin administration, even with removal of gonadal steroids via castration [57]. However, the changes in melatonin binding observed in the song-control nuclei over the course of a year are not directly correlated to changes in nuclei volume [58]. Also, reproductive state does not determine melatonin binding in these sites, for photostimulated male European starlings housed in semi-natural environments do not downregulate 2-[^125^I]iodomelatonin binding in Area X, as expected in starlings housed in the laboratory [58]. These differences in 2-[^125^I]iodomelatonin binding can result from synergistic variables offered by semi-natural environments that are absent from laboratory settings [59]. There are also ontological differences (in zebra finch, *Taeniopygia guttata*) [60] and sex differences for melatonin binding in the avian brain (in house sparrow, *Parus major*) [61]; in quail, *Coturnix japonica*) [62]; in starling, *Sturnus vulgaris*) [58]. It is important to consider this intraspecies variation for comparative research in melatonin binding (e.g., melatonin binding in avian and testudine brains [63]).

Radiolabeled ligand studies provide integral information to localize melatonin-binding sites in the brain and ascertain the density of melatonin receptors. The extent to which a given neural site binds melatonin depends on the time of day, season, and housing condition at the time of tissue collection. Sequencing and cloning techniques optimally parse out the different types of melatonin subtype receptors.

### 3.2. Melatonin Receptor Subtypes

Melatonin’s direct and indirect effects on reproduction are contingent upon its action as a hormone that binds particular subtype receptors [43]. Differences in regulatory functions of melatonin-binding proteins have procured categories of melatonin receptor subtypes. Melatonin subtype receptors include membrane-bound G-protein coupled receptors and nuclear orphan receptors. Research on the functional role of melatonin receptors tends to focus on the membrane receptors, likely due to their higher affinity and specificity for melatonin. However, there is evidence that nuclear orphan receptors that directly bind melatonin might be primary targets downstream in the membrane receptor signalling pathway [64]. Based on localization and administration studies, functions for melatonin membrane receptors likely regulate metabolic, cardiovascular, immune, and reproductive systems (for review see [65]).

**Figure 3:**
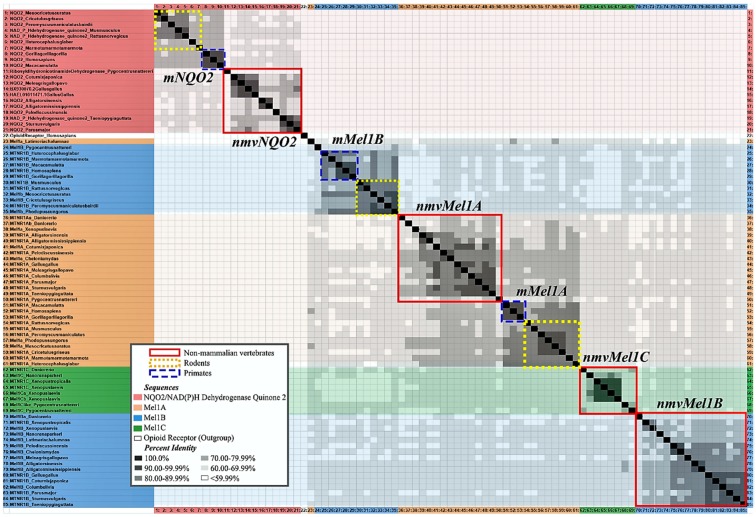
Percent Identity Matrix of Melatonin Membrane Receptor Subtypes and Quinone Reductase 2. Analysis run by Clustal Omega 2.1 using NCBI GenBank. Red solid lines outline non-mammalian vertebrates (***nmv***), yellow dotted lines outline rodents, and blue dashed lines outline primates. Shades of grey indicate ranges of percent identity, black 100% and progressively lighter shades of grey down to 60%. Values <60% identity are white. Non-mammalian vertebrate (***nmv***) Mel1B sequences (lower right corner) do not have significant similarity with mammalian (***m***) Mel1B sequences (mid-upper left), suggesting that these are phylogenetically distinct melatonin membrane receptors. There is higher similarity within Mel1A receptor subtypes across vertebrates (mid-lower right). The quinone family in the upper-left [named NAD(P)H dehydrogenase quinone 2, ribosyldihydronicotinamide dehydrogenase, or NQO2] shows no significant similarity with other membrane melatonin receptor subtypes with percent identity across vertebrates and melatonin receptor subtypes <60%, supporting the position that QR2/NQO2 is not a putative MT3 membrane subtype receptor.

Research on melatonin receptor subtypes has its challenges. Autoradiography shows 2-[^125^I]iodomelatonin binding density but fails to distinguish melatonin receptor subtypes. Furthermore, nomenclature for melatonin subtype receptors varies across species and publications. Overall, research in mice and rats uses MT1 and MT2, but there are exceptions for some rodents, and Mel1a and Mel1b are used instead. MT3 is considered a mammalian melatonin receptor subtype as well, characterized as the enzyme quinone reductase 2 (QR2 or NQO2) [66]. The discovery of a binding site for melatonin on QR2 justified its appellation of “MT3” as a putative melatonin membrane receptor [67]. The functional roles of MT1 and MT2 might not be analogous to Mel1a and Mel1b, respectively, in other non-mammalian vertebrates. Ideally, nomenclature is not conflated without comparative molecular evidence. Using the Percent Identity Matrix by Clustal Omega Multiple Sequence Alignment [68], Figure 3 compares mRNA sequences for Mel1a/MT1 and Mel1b/MT2. Furthermore, it shows MT3 does not share significant (>60%) percent identity with mRNA sequences of other melatonin membrane receptors, supporting pharmacological evidence that it is not a melatonin receptor. This finding will be discussed later in this section on melatonin receptor subtypes.

Non-mammalian vertebrate research consistently uses Mel1a, Mel1b, and Mel1c for nomenclature. For the purpose of this review, the names are kept consistent with their use in the cited primary literature, but this is not to imply the Mel1a/MT1, Mel1b/MT2, or Mel1c/putative MT3 are interchangeable. While there is evidence of some shared identity of melatonin subtype receptors across vertebrates (Figure 3), the names are assigned based on pharmacological binding properties, such as affinity and specificity. These binding properties have interspecies variation in subcellular regulation and differ across tissue types [69,70]. In some vertebrates, gene polymorphisms and phylogenetic analyses of melatonin subtype receptor amino acid sequences were studied [71]. The percent identity matrix presented here (Figure 3) is an alignment of mRNA sequences and includes QR2/NQO2, the putative MT3 receptor.

**Figure 4:**
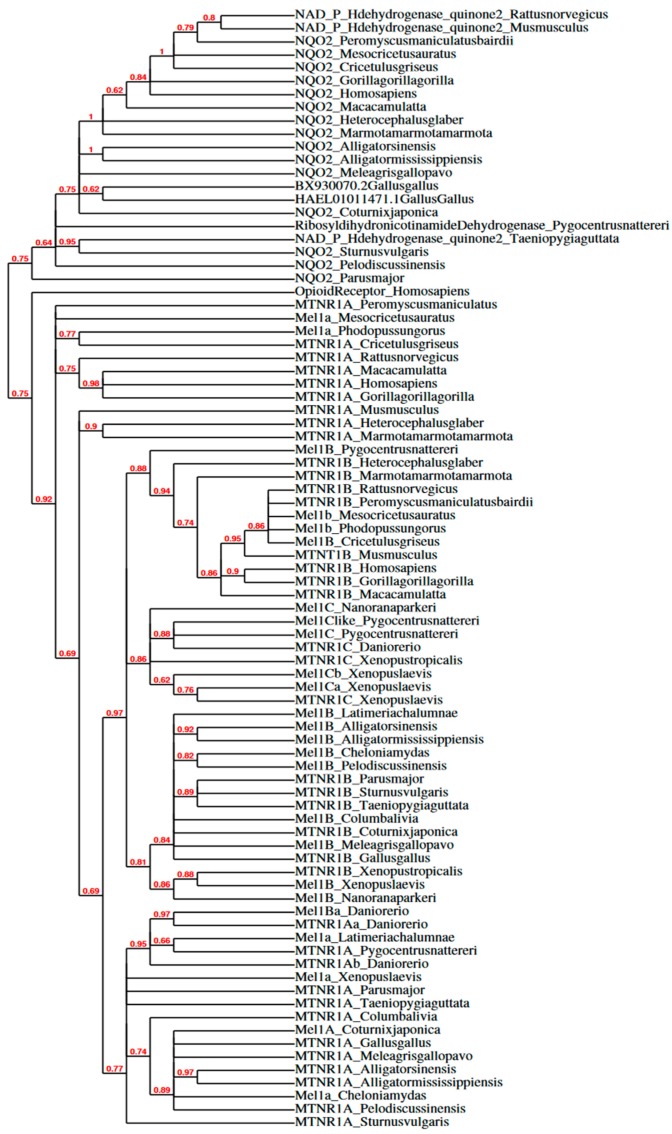
Cladogram of melatonin membrane receptor subtypes and quinone reductase 2. Modified from neighbor-joining tree (without distance corrections), generated by *Phylogeny.fr* (Dereeper et al., 2008 & 2010). Red numbers represent branch support values. Branch support values smaller than 50% are collapsed. The quinone family is named NAD(P)H dehydrogenase quinone 2, ribosyldihydronicotinamide dehydrogenase, or NQO2, based on how it is named in the NCBI database. Opioid Receptor for *Homo sapiens* (NCBI Accession No. L29301.1) served as the outgroup. NCBI Accession Numbers and full names of sequences are in Figure 4. The mRNA sequences of non-mammalian vertebrates (***nmv***) ***Mel1A*** diverged more recently than mammalian (***m***) ***Mel1B*** from ***mMel1A***. The pharmacological evidence that Mel1A/MT1 in birds has one order of magnitude lower affinity for 2-[^125^I]iodomelatonin than MT1 in some mammals (rabbit, sheep and horse) and one order of magnitude higher than MT2 other mammals (Syrian and Siberian hamsters) supports the phylogenetic evidence presented here that avian Mel1A mRNA is not evolutionarily homologous with mammalian Mel1A/MT1 mRNA.

Besides binding melatonin, receptor subtypes share few conserved traits across vertebrates. The localization, activation, and regulation of receptor subtypes vary widely. Future studies should isolate and determine the affinity, specificity, and stability of melatonin and other agonist/antagonist binding for receptor subtypes in species who diverged further back in evolutionary history. A phylogenetic tree of melatonin subtype receptor amino acid sequences was previously created [71], and Figure 4 shows a cladogram by *Phylogeny.fr* [72,73] of melatonin subtype receptor mRNA sequences from NCBI GenBank (Table 1 shows the list of NCBI Accession Numbers). Melatonin membrane receptors discussed here include research in mammals, birds, and other non-mammalian vertebrates, specifically elucidating the functional role of melatonin membrane receptor subtypes in the brain and gonads (for studies elucidating melatonin subtype receptors in peripheral tissues in mammals, see [44,67,74] and in birds see [75,76,77,78].

### 3.3. In Mammals

What regulates melatonin membrane receptor subtypes, and what does activation of different subtypes subsequently regulate in mammals? Each subtype receptor appears to have unique roles in different tissues and species. Methods used to isolate MT1, MT2, and the putative MT3 receptor are reviewed here.

Most mammalian Mel1a and MT1 research has focused on melatonin-binding in the suprachiasmatic nucleus (SCN) (for review of applications, see [79]). As in most biomedical research, transgenic mice are used for their slight photoperiodicity and genetic homogeneity. In C3H/HeN mice, *mt1* mRNA and MT1 protein can be detected in the SCN, and there is evidence to suggest that this melatonin subtype receptor is regulated by diurnal and circadian mechanisms [54]. In C3H/HeN mice housed in constant darkness for 6 weeks, a peak in the density of 2-[^125^I]iodomelatonin binding in the SCN was observed at the beginning of the subjective day determined by free-running activity patterns [54]. For C3H/HeN mice housed in light-dark cycles, low *mt1* mRNA levels were measured from SCN tissue collected during the day, and *mt1* mRNA expression peaked at the beginning of the dark period, coincident with increases in circulating melatonin [54]. Because melatonin binding peaked in the SCN approximately 8 h after the peak in *mt1* mRNA expression, it appears that melatonin receptor mRNA and protein are differentially regulated in C3H/HeN mice [54]. Ultimately, studies in different strains of mice revealed that melatonin receptor MT1 is necessary and sufficient for transmitting the photoperiodic signal [80]. There is the option of using “nature’s knockout” to study Mel1a, for the Siberian hamster (*Phodopus sungorus*) Mel1b gene has nonsense mutations in the coding region [81]; however, there is still a Mel1b sequence stored in NCBI GenBank for *P. sungorus* (Accession Number U57555.1). Using this model organism and administering MT1/MT2 receptor agonist, Prendergast determined MT1 was necessary and sufficient to transduce photoperiodic information and alter reproductive and metabolic physiology [82]. The SCN in Syrian hamsters (*Mesocricetus auratus*), meanwhile, did not show 2-[^125^I]iodomelatonin binding and was assumed to not have melatonin receptors. However, a study on post-natal (PN) Syrian hamsters revealed that Mel1a binding and expression was present in the SCN and highest before PN 8. While SCN binding of melatonin plummeted after PN 8, the expression levels of *Mel1a* mRNA decreased but not as significantly as autoradiographical binding, implying the developmental regulation of melatonin receptors in the Syrian hamster SCN is post-transcriptional [83]. Thus, we cannot disregard the effects of aging on melatonin receptor regulation in general.

Research on melatonin receptor subtypes becomes more complex when considering MT1 and MT2 together. In 1995, the early stages of research on melatonin subtype receptors (at the time, named ML1 and ML2) were outlined by Dubocovich [84]. Similarities and differences in the peripheral functions of MT1 and MT2 in mammals have been more recently revisited by Dubocovich and Markowska in 2005 [67]. Several steps were taken to distinguish these membrane melatonin-binding receptors. Firstly, pharmacological and functional characteristics of these two receptor subtypes are distinct [85,86]. If the binding is stable, saturable, reversible, and specific, the ligand affinity of the receptors can be tested to determine if they are, in fact, distinguishable subtypes. Secondly, a specific radioligand was discovered to selectively target melatonin ML2 receptor across tissues in rodents [87]. Antagonists with a higher specificity for MT2 were used to isolate and distinguish the functional role of MT2 from MT1 receptor subtypes. Blocking specific subtype receptors corresponded to downstream effects on behavior (e.g., activity rhythms and anxiety tests). For example, in C3H/HeN mice, 4-phenyl-2-propionamidotetraline (4P-PDOT) blocked melatonin-mediated phase advances in circadian rhythms [88]. In rats, luzindole blocked melatonin-induced antinociception [89]. However, luzindole also functions as an antagonist for MT1 receptors, so the receptor-mediated effects observed in [89] can include MT1. Another experiment, describing luzindole as a nonselective antagonist to MT1/MT2, observed luzindole and 4P-PDOT could block melatonin-induced phase advances in the SCN of Long-Evans rats when administered independently [90]. Because 4P-PDOT has a higher specificity for MT2 [88], the use of 4P-DOT blocked activation of MT2 in the SCN and prevented phase advances in circadian activity rhythms of mice and rats. Luzindole also had antidepressant effects on C3H/HeN mice subjected to the forced swim test [91]. These changes in behavior resulted from selectively targeting melatonin subtype receptors with specific antagonists.

Other methods used to distinguish specific functional roles of melatonin subtype receptors include western blots and southern hybridization, which localized MT1/MT2 in peripheral tissues [74]. The limited distribution of MT2 protein in mice (restricted to the brain and lung) compared to the peripheral distribution observed of MT1 protein (including the brain, lung, heart, liver, and kidney) suggests a distinct functional role for MT2 in these tissues [74]. It should be noted, however, that MT1/MT2 mRNA expression using RT-PCR method showed low expression of MT2 in the rat liver and heart [92]. Either differences in species and methods affected results, or there is differential transcriptional/translational regulation of MT1 and MT2 in rodents.

Lastly, selectively bred rodents are ideal for parsing out the distinctions between MT1 and MT2 subtype expression and regulation. In situ hybridization and RT-PCR show that targeted disruption of *Mel1a* in selectively bred C57BL/6 mice disrupted 2-[^125^I]iodomelatonin binding in the brain, suggesting Mel1a represents 99% of binding observed under this particular protocol [93]. However, C57BL/6 mice with disrupted *Mel1a* are still capable of phase-shifting, so it was conjectured that relatively low levels of Mel1b compensates [93]. By using MT1-KO and MT2-KO mice, another study deduced the antidepressant effects of luzindole were mediated through the MT2 receptor [94]. MT1KO C57BL/6 mice have revealed the connection between the MT1 subtype and depressive or anxiety-like behaviors [95]. In summary, transgenic mice provide a useful model alongside melatonin receptor antagonists to distinguish the functional roles of MT1 and MT2 in rodents (for review of targeted deletion of melatonin receptor subtypes, see [96]). Mice models have been developed for studying therapeutic applications of selective blocking and activation of melatonin subtype receptors [97]. However, transgenic mice are not representative of photoperiodic breeding mammals. Research on the effects of melatonin receptor subtype knock-outs on seasonal reproductive timing should be conducted in other species.

Rodents that are considered more heterogenous than transgenic strains of mice can be used to effectively study melatonin for more ecologically relevant questions. White-footed mice (*Peromyscus leucopus*) from Connecticut and Georgia are sensitive and insensitive to melatonin, respectively [49]. A longer duration of melatonin is observed in *P. leucopus* housed in short photoperiods [98]. While maintaining short photoperiods (8L:16D) for long-term housing of *P. leucopus* mice from Connecticut, Georgia, and Maine, only mice from Georgia remained reproductively competent [99]. Meanwhile, mice from Connecticut and Maine underwent testicular regression and spontaneous recrudescence within this extended exposure to short photoperiods [99]. Daily injections of 50 µg of melatonin in wild-caught mice from Connecticut and Georgia led to six out of fourteen mice from Connecticut molting into winter pelage with no observable effects on mice collected from Georgia [100]. In *P. leucopus* mice wild-caught and selectively bred from Virginia, strains that were responsive and nonresponsive to changes in photoperiod were studied for differences in melatonin binding [101]. Selectively bred, nonresponsive white-footed mice showed higher 2-[^125^I]iodomelatonin binding in the medial preoptic area (mPOA) and nucleus stria terminalis, which might be due to differences in density or affinity of receptors in these areas [101]. These findings suggest that intraspecies geographical variation in melatonin sensitivity is fixed in the wild.

Seasonal changes in melatonin responsiveness is accompanied by daily changes of pineal synthesis and secretion of melatonin, and these fluctuations can be regulated by melatonergic negative feedback. Circulating levels of melatonin increase with the onset of darkness. However, this elevation in melatonin concentration is temporally constrained and not directly related to the absence of light. There is a detectable decrease in circulating melatonin levels before the onset of dawn, or before light directly inhibits melatonin synthesis. This implies that melatonin is regulated by something other than light during this pre-dawn trough. Specific melatonin receptor antagonists (luzindole and 4-P-PDOT) were administered to white-footed mice [102]. The MT1/MT2 antagonist, luzindole, prevented the light-independent drop in plasma melatonin typically observed late night/early morning while it was still dark [102]. This was not the first evidence to suggest lagging, homeostatic regulation of melatonin on itself via its own receptors. Melatonin affects MT2 functionality to regulate tissue sensitivity to the melatonin signal in rats as well. When administered at physiologically relevant concentrations and durations, melatonin desensitized MT2 in the rat SCN by preventing stimulation of PKC [103], providing yet another mechanism by which melatonin regulates the circadian clock through melatonin receptor subtypes. The potential for negative feedback regulation of pineal melatonin via binding and activation of melatonin subtype receptors MT1 and/or MT2 is an exciting possibility worth further investigation in other vertebrates. However, it is important to note that 2-[^125^I]iodomelatonin binding was not observed in the pineal complex of fifteen avian and three testudine species previously studied [63], and as described in mammals, binding properties of receptors can vary based on age, reproductive state, and the time of day the animal was used in the experiment.

There is relatively less research on the functional role of the putative mammalian MT3 receptor, part of the quinone reductase enzyme family and known as quinone reductase 2 (QR2) [69,104]. QR2 is an enzyme and not a classical seven transmembrane domains receptor [105]. MT3 has been described as the Syrian hamster homologue of human QR2 (95% identity) based on amino acid sequencing [106]. Prazosin was used as an MT3 antagonist [89], and 5-MCA-NAT was used as an MT3 agonist in rabbits [107] and monkeys [108]. However, nuclear magnetic resonance studies found no evidence to suggest melatonin is a substrate at all for MT3 [109], suggesting that melatonin functions in the capacity of an antioxidant in conjunction with QR2. Despite this experiment, there are still countless publications referring to QR2 as the “putative MT3 receptor”. 5-Methoxycarbonyl-amino-*N*-acetyltryptamine (MCA-NAT), a partial agonist of MT1 and MT2 at sub-micromolar ranges, does not elicit any detectable receptor-like responses from Chinese Hamster Ovary (CHO) cells overexpressing quinone reductase 2 [110], even though MCA-NAT was used to study molecular responses to melatonin in chick retinal development [111] and bovine blastocysts [112]. These studies assume that MCA-NAT specifically targets MT3, and luzindole non-selectively blocks MT1 and MT2. These assumptions, based on the premise the MCA-NAT targets MT3 and luzindole blocks MT1/MT2, ascribe the observed physiological effects to the functional role of MT3. Given insufficient evidence of MCA-NAT binding QR2, and contrasting evidence that QR2 even binds melatonin, studies that assume QR2 is functional melatonin subtype receptor should be reviewed with skepticism.

The inconsistent nomenclature QR2 in NCBI GenBank includes and is not limited to NAD(P)H quinone dehydrogenase 2, ribosyldihydronicotinamide dehydrogenase [quinone], and NRH: quinone oxidoreductase 2. The most frequently used acronym for QR2 identified for genes included in the phylogenetic analysis conducted here is NQO2 (see Figure 2 and Figure 3). Despite persisting claims of ambiguity on the matter (Dubocovich & Markowska, 2005), the percent identity matrix shown here has no significant overlap with the mRNA sequences for NQO2 and mRNA sequences for membrane melatonin receptor subtypes (Figure 2). This mRNA phylogeny supports pharmacological evidence (Boutin et al., 2008) that QR2/NQO2 is not a membrane melatonin subtype receptor.

Most melatonin receptor research in mammals focuses on the brain or eyes. Here, an overview of MT1/MT2 in the gonads of rodents shall be discussed. The antioxidant role of melatonin in ovaries was reviewed previously [113], but the presence of melatonin receptors in ovarian tissue suggests melatonin plays an endocrine role as well. In rat ovaries, PCR and in situ hybridization showed *mt1*/*mt2* expression at various stages of the estrous cycles [114]. The functional relationship between melatonin subtype receptors and estrous cycles is unexplored to date. In future studies that assess the physiological effects of exogenous melatonin in the ovaries, concentrations of melatonin administered must be considered. Supraphysiological levels of melatonin administered to the Chinese hamster ovary (CHO) cell line increased MT1 detected and decreased affinity observed through competitive binding with 2-[^125^I]iodomelatonin [115]. These melatonergic effects on MT1 in the CHO cell line can be mediated through specific modifications of the subcellular signalling cascade [116]. However, melatonin administered at physiologically relevant levels had no effects on CHO cell line MT1 [115]. The role of melatonin in testes on sperm production was studied across several groups of mammals. While it was previously observed that 2-[^125^I]iodomelatonin did not bind in the gonads of mammals other than shrews (family Soricidae) [117], regulatory mechanisms of MT1/MT2 expression were later found in spermatozoa and ejaculate of five different breeding types of mammals [118]. Melatonin’s endocrine role in mammalian testes appears to, in part, regulate sperm maturation. In rat testes, *mt1* and *mt2* are expressed throughout development [119], and melatonergic effects on rat spermatogenesis and steroidogenesis were studied [120,121,122,123]. The next section overviews melatonin receptor subtypes in birds.

### 3.4. In Birds

The majority of reviews on melatonin subtype receptors focus on mammals. This section provides a comprehensive review of melatonin subtype receptors in birds. Early work focuses on the general distribution and characterization of 2-[^125^I]iodomelatonin binding in the brain of chicks [124] and quail [125]. The affinities and densities of 2-[^125^I]iodomelatonin binding observed in duck, goose, pigeon, and turkey [46] were an order of magnitude lower than what was previously described in quail [125], suggesting that the pharmacological properties of melatonin receptors might not be conserved across different species of birds. 2-[^125^I]iodomelatonin binding also was compared in brains collected from five orders of birds (Psittaciformes, Passeriformes, Columbiformes, Galliformes and Anseriformes) and turtles, and melatonin binding was not observed in the pineal, adenohypophysis, or tuberal hypothalamus (analogous to SCN) in any of the avian or testudine species studied [63]. Additionally, our understanding of melatonin binding in the avian brain was challenged by the discovery of how photoperiodic history affects 2-[^125^I]iodomelatonin binding in quail [126] and songbirds [56,58]. 2-[^125^I]iodomelatonin binding densities in the brain varied based on photoperiodic history, reproductive state, and sex of birds [62].

Parsing out different subtype receptors in the avian brain is useful for understanding context-dependent differences. Melatonin receptor antagonists such as prazosin and luzindole selectively block specific subtypes in broiler chickens [127]. However, the affinity and specificity of these antagonists can not be guaranteed, especially considering previous work found differences in avian melatonin receptor pharmacology in different species [46]. Furthermore, expression analysis of subtype receptor sequences identifies the presence of mRNA in different neural sites. RT-PCR was used to identify Mel1a, Mel1b, and Mel1c in the chick brain [128]. The same technique was used in zebra finch brain and peripheral tissues and found significant rhythms of both *Mel1a* and *Mel1b* expression in cerebellum, diencephalon, retina, and tectum opticum [77]. *Mel1a* expression patterns showed significant rhythms in the telencephalon, and *Mel1b* showed significant rhythms in the pineal gland [77]. However, the expression patterns of *Mel1c* in the zebra finch brain did not appear to be significantly rhythmic [77]. In situ hybridization assesses the distribution of specific melatonin subtype receptors in the avian brain (in quail [129] and in blackcap and zebra finch [130]). These findings provide some insight into the specific functional roles of melatonin subtype receptors in birds. For instance, *Mel1c* expression was co-localized with gonadotropin inhibitory neurons (GnIH) [129]. Several neural sites involved in sensory motor integration also co-express *Mel1a* & *Mel1b* or *Mel1a* & *Mel1c*, with few sites expressing all three subtypes or *Mel1b* & *Mel1c* [130]. In male zebra finches (*Taeniopygia guttata*), the song motor control pathway expressed melatonin receptor subtypes, and administration of Mel1b antagonist (S20928) transiently shortened the length of the song [131]. Considering inter-species differences in melatonin subtype receptor characteristics previously described, the affinity and specificity of S20928 for the Mel1b receptor still needs to be determined in this species.

Melatonin subtype receptors have also been studied peripherally in birds. In chicken, melatonin subtype receptor mRNA temporal patterns and spatial distribution were found in the retina [76] and in the spleen [78]. In the latter study, age-related changes in subtype receptor expression were discovered [78], indicating age in birds, as previously described in mammals, is an important variable to account for in future studies.

Melatonin receptor subtypes in avian gonads has exciting implications for the endocrine role of melatonin in the avian HPG axis. 2-[^125^I]iodomelatonin binding was observed in the testes and ovaries of chicken, duck, and quail [117]. Partial sequences of melatonin subtype receptors from the chicken ovary were identical to subtype receptor sequences from the brain [132]. Follicles at varied stages of development expressed different levels of melatonin subtype receptors. Small white follicles only expressed *Mel1b*, and small yellow follicles expressed all three subtypes [132]. *Mel1a* was restricted to the chicken thecal layer while *Mel1b* and *Mel1c* were expressed in both chicken granulosa and thecal layer [132]. In European starlings, we previously found *Mel1b* and *Mel1c* expression correlated with expression of gonadotropin inhibitory hormone (GnIH) and its receptor (GnIHR), respectively [133]. Furthermore, we found *Mel1b* expression in starling testes appeared to correspond with daylength and *Mel1c* with reproductive state, suggesting these receptor subtypes are differentially regulated and serve different functions in songbird testes [133]. There is also differential photoperiodic regulation of melatonin subtype receptor expression in tropical bird testes (*Perdicula asiatica*, see [134]). The effects of monochromatic light on ovarian melatonin subtype receptor expression were studied in chickens [135]. Hens that were housed in red (660 nm) light expressed *Mel1a* and *Mel1c* at significantly higher levels than all other groups [135]. Furthermore, hens housed in blue (480 nm) light laid significantly more eggs than all other groups [135]. Blue light suppressed pineal melatonin synthesis in chickens [136,137]. There is an inverse relationship between fecundity and melatonin subtype receptor expression in the chicken ovary [135]. The implications of these findings are discussed in greater detail in the section of this introduction on melatonin signalling.

Methods used in mammals to study melatonin subtype receptor functionality, such as transgenics [96,97] and natural variation in intraspecies melatonin sensitivity [49], have no obvious equivalents in birds. Mammalian melatonin receptor agonists or antagonists show variance in how they bind (i.e., specificity and affinity) based on the age and sex of the animal as well as the time of day of administration. To account for these variables across avian species would be an exhaustive undertaking. There are no established practices for taking advantage of natural variation in melatonin sensitivity within a species of bird found in the wild, as previously described in white-footed mice. Gene editing technologies, such as CRISPR-Cas [138,139], provide a novel approach for melatonin subtype receptor research. Comparative research on melatonin receptor subtypes in other non-mammalian vertebrates can inform future experiments in birds, and the next section provides an overview of emerging research in this area.

### 3.5. In Other Vertebrates

The effect on pineal extractions on skin pigmentation of *Xenopus* tadpoles was discovered a century ago this year [140]. The pineal complex and melatonin were causally connected to the diurnal rhythms of color change in lamprey much later (*Lampetra*) [141]. Forty years after McCord and Allen (1917) published their findings in *Xenopus*, melatonin was isolated [142], and nearly thirty years later, the relationship between melatonin and photoperiod and their effect on anuran larval development was empirically confirmed [143]. Despite this long history investigating the effects of melatonin on amphibia, research on melatonin receptor binding and subtypes in non-mammalian and non-avian species is relatively recent. 2-[^125^I]iodomelatonin binding was studied in amphioxus (*Branchiostoma lanceolatum*), Atlantic hagfish (*Myxine glutinosa*), larval and adult lamprey (*Petromyzon marinus*), little skate (*Raja erinacea*), and rainbow trout (*Oncorhynchus mykiss*), and all but hagfish showed specific binding in the brain of these species [47]. In vitro culture of the pineal complex from lamprey (*Petromyzon marinus*) showed fluctuations in melatonin secretion when kept in light:dark (12L:12D) cycles, and this rhythm did not persist in constant darkness [144]. Melatonin secretion from the lamprey pineal complex is likely temperature dependent because the rhythm of melatonin secretion from the cultured lamprey pineal complex was maintained in 20 °C but abolished in 10 °C in constant darkness [145], showing that melatonin secretion is regulated by more than light in other vertebrates. In turtles (*Chrysemys picta*), 2-[^125^I]iodomelatonin binding was observed primarily in the visual system [146], potentially affecting photosensitivity and the capacity for light to affect melatonin synthesis.

Melatonin receptor subtypes, however, could not be confirmed until sequencing technologies became more accessible. *Mel1c* in *Xenopus* was cloned just over two decades ago [85], around the same time mammalian *Mel1a* and *Mel1b* were cloned [86]. In the past 10 years, melatonin receptors were cloned and sequenced from sea bass (*Dicentrarchus labrax*) [147], sole (*Solea senegalensis*) [148], and the mudskipper (*Boleophthalmus pectinirostris*) [149]. The study in sole showed seasonal and daily fluctuation in expression levels of melatonin subtype receptors [148], and melatonin subtype receptors in the mudskipper seemed to synchronize with the semilunar spawning rhythm [149]. In the velvet belly lantern shark (*Etmopterus spinax*), melatonin stimulated light production by isolated photophore-filled skin patches [150]. Luzindole and 4P-PDOT blocked this response, suggesting shark luminescence is mediated by the MT2 receptor [150]. Research on melatonin and its receptor subtypes in aquatic craniates and poikilotherms extends beyond pure classification and is answering questions related to the functional roles of melatonin receptor subtypes (for review, see [151]).

Recent research on the functional role of membrane melatonin receptors in Actinopterygii focused on reproductive and lunar cycles. 17α,20β-dihydroxy-4-pregnen-3-one (DHP) is used as a biomarker for the onset of spawning in mudskippers, and melatonin injections increased DHP in vivo and in cultured ovaries [149]. Melatonin subtype receptor expression correspond with the lunar cycles and with the spawning season in mudskippers [149]. However, it is important to note only one reference gene (*β-actin*) was used in the qRT-PCR analysis [149], and accurate normalization ideally includes multiple control genes [152]. In the orange-spotted grouper (*Epinephelus coioides*), *mt1* and *mt2* expression in the brain varied based on reproductive state [153]. Again, only one reference gene (*18S*) was used in the qRT-PCR analysis [153]. In the gold-lined spinefoot (*Siganus guttatus*) fluctuations in *MT1* and *Mel1c* expression corresponded to lunar brightness [154]. The overall relationship between lunar brightness, melatonin, and fish reproduction was previously reviewed [155]. Cultured pineal of golden rabbitfish (*Siganus guttatus*) varied in melatonin content based on exposure to moonlight intensity [156]. In the grass puffer (*Takifugu niphobles*), *mel1a* (1.4 & 1.7), *mel1b*, and *mel1c* appear to be expressed in constant darkness with ultradian regulation in the pineal gland, so there might be light-independent lunar oscillations [155]. Given these results show lunar patterns in tissue cultured in darkness, there is more to lunar cycles than moonlight affecting melatonin subtype receptors in fish, possibly relating to the type of environment in which the fish are found in the wild (fresh water, salt water, tidal patterns, still water, etc.).

Comparative research on melatonin subtype receptors has broader implications across vertebrates. Findings connecting moonlight to melatonin synthesis in mammals are comparable to lunar patterns observed in fish. Moonlight suppressed pineal melatonin production in Syrian hamsters [157]. However, moonlight appears to have no effect on pineal AANAT activity and melatonin content in the cotton rat (*Sigmodon hispidus*) [158], reminding us that moonlight will not show conserved effects across species or individuals. The effects of moonlight in fish might be more conserved since the pineal itself is photoreceptive in this order. Whether the animals were caught in the wild or reared in the laboratory is also cause for such variance (for review, see [59]). The effects of moonlight on avian melatonin synthesis has yet to be investigated. Given that (1) the pineal in birds is photoreceptive, (2) the mRNA sequences of melatonin subtype receptors in birds and other non-mammalian vertebrates are relatively conserved (see Figure 2 and Figure 3), and (3) urban light pollution at night affects melatonin content in birds, even at low levels (in European blackbirds, *Turdus merula* [8] and in western scrub-jays, *Aphelocoma californica* [159]), we should consider the potential for moonlight to affect melatonin and its subtype receptors in birds as previously observed in fish.

### 3.6. Summary

The determined location of melatonin-binding sites and quantified expression levels of melatonin receptor subtypes set the conditions of possibility for melatonergic functionality in reproductive physiology. Given that binding densities revealed by autoradiography do not isolate specific melatonin subtype receptors, we must use protein assays and RNA sequencing to determine the presence of melatonin subtype receptors in a given tissue. The multiple sequence alignment of amino acid sequences previously determined shared identities of melatonin subtype receptors across a subset of vertebrates [71,86,147,148,149]. Since NCBI GenBank offers an expansive list of melatonin subtype receptor mRNA sequences from more species now than ever before (see names of sequences and Accession No. organized in Table 1), the need for a multiple sequence alignment and phylogenetic analysis of mRNA sequences from known and putative melatonin subtype receptors in vertebrates was fulfilled here (see Figure 2 and Figure 3). Previous research showed amino acid sequences of melatonin membrane receptors shared distinct clades, but this analysis was limited to alignment of amino acid sequences from four mammals, one fish, one bird, and one frog [128]. The mRNA multiple sequence alignment conducted here includes 11 mammals, 7 birds, and 10 other vertebrates in Mel1A and a subset of this list for available sequences for other subtypes (for Accession No. see Table 1), rendering the most extensive multiple sequence alignment of melatonin subtype receptor mRNA sequences to date. Within the percent identity matrix (Figure 2, analyzed with Clustal Omega), mRNA sequences of melatonin receptor subtypes in Aves share a higher identity with sequences from Osteichthyes, Testudines, Crocodilia, Anura, and Latimeria (~70.00–99.99%) than with Mammalia (~60.00–79.99%). The cladogram (Figure 3, analyzed in *Phylogeny.fr*) shows higher parsimony with non-mammalian melatonin subtype receptors (***nmvMel1A***, ***nmvMel1B***, ***nmvMel1C***) and mammalian Mel1B (***mMel1B***) than with mammalian Mel1A (***mMel1A***). This suggests that mammalian Mel1A (***mMel1A***) and ***nmvMel1A*** are not homologous. Furthermore, the clade of QR2/NQO2, the putative MT3 receptor, diverged further back from the clade of melatonin subtype receptors than the outgroup (*Homo sapiens* Opioid Receptor, Accession No. L29301.1, selected based on rat µ Opioid R outgroup used in [128]). Because the identity of QR2/NQO2 is less than 59.99% with all membrane melatonin subtype receptors across vertebrates (Figure 2), the findings of this matrix support pharmacological evidence [109] that QR2/NQO2 is not a melatonin membrane subtype receptor.

In addition to quantifying mRNA sequences and localizing melatonin subtype receptors, future studies must rigorously test the binding properties of agonists and antagonists to determine affinity and specificity with melatonin subtype receptors. Binding properties of melatonin subtype receptors not only vary across species but also can vary with time of day, age, and sex. Ligands that target melatonin receptor subtypes in one species does not have the same affinity and specificity in another species. Studies that do not test the binding affinities of melatonin receptor agonists/antagonists in the model organism being used can not be certain that the observed physiological and behavioral changes are resulting from targeted activation or blocking, convoluting melatonin subtype receptor applications and therapies. Following melatonin binding to specific subtype receptors, physiological changes that occur in the context of reproduction will be addressed in the next section on melatonin signalling.

## 4. Melatonin Signalling in Reproduction

“To think that we understand survival value completely ... is to think that, once it is obvious that sex hormones control mating behaviour, we need not inquire into the way they do this, nor into the interaction between various endocrine processes that are involved” [160].

The classical definition of a hormone is a chemical that is synthesized and secreted from a particular gland into circulation, binds to a specific receptor, and induces a physiological change. Distinguishing the endocrine function of melatonin from its role as an antioxidant is more complex than it first may seem. While the main specialized gland for melatonin synthesis is considered to be the pineal, removing this gland does not remove all circulating melatonin. The four enzymes that synthesize melatonin from the precursor tryptophan are expressed in several other tissues, strongly suggesting that melatonin synthesis is distributed peripherally (see Melatonin Synthesizing Enzymes). Furthermore, melatonin endocrine action can be mediated by melatonin subtype receptors, but melatonin and its precursor *N*-acetylserotonin also can function as free-radical scavengers as part of an antioxidant cascade, and their oxidized byproducts are antioxidants. To differentiate the classical endocrine role of melatonin from its antioxidant effects requires us to focus on receptor-mediated functions, and yet unless specific melatonin receptor agonists/antagonists are administered, receptor-mediated functions cannot be isolated.

This section addresses physiological responses induced by melatonin in the context of seasonal reproduction. It is important to note that many of the studies reviewed here do not use specific melatonin receptor agonists/antagonists, so whether melatonin corresponds to specific reproductive physiological changes in its capacity as an antioxidant or hormone is inextricable unless the study determines if specific subtype receptors are expressed or immunolabeled. Approaches that are commonly employed to study melatonin signalling in seasonal reproduction include (1) ablation, lesion, or culture of melatonin-synthesizing and/or binding sites; (2) co-localization of melatonin-synthesizing and/or binding sites within neural and/or peripheral reproductive pathways; and (3) exogenous administration of melatonin. This section focuses on studies that employ these approaches to study the physiological relevance of melatonin in vertebrate reproductive pathways with the hypothalamo-pituitary gonadal (HPG) axis as the point of convergence.

### 4.1. HPG Axis

Among the various endocrine feedback loops fluctuating within age- and sex-dependent homeostatic ranges is the hypothalamo-pituitary gonadal (HPG) axis. A subset of hypothalamic neurons synthesize and transport gonadotropin releasing hormone (GnRH, or luteinizing-hormone-releasing-hormone, LHRH, see [161]). There are several different subtypes of GnRH across vertebrates (for reviews, see [162,163,164]), and these GnRH subtypes have yet to be studied in conjunction with melatonergic effects on seasonal reproductive timing in vertebrates. In mammals, kisspeptin (*Kiss*) and RFRP (or gonadotropin inhibitory hormone, GnIH) are hypothalamic neuropeptides that, respectively, stimulate and inhibit the synthesis and release of GnRH (for reviews on kisspeptin, see [165,166] and for GnIH/RFRP, see [167,168,169]).

Pulsatile secretion of GnRH binds the anterior pituitary and releases gonadotropins luteinizing hormone (LH) and follicle-stimulating hormone (FSH) (for review, see [170]) in a frequency-dependent fashion [171]. Gonadotropins can also be regulated by other factors, such as inhibins (for review, see [172]). GnIH also binds in the pituitary and decreases gonadotropin synthesis and release [173]. LH and human chorionic gonadotropin (hCG) both bind the same receptor, but they are differentially regulated and support different aspects of the reproductive cycle (for review, see [174]). Upon synthesis and release from the pituitary, LH and FSH bind receptors in the gonads (testes/ovaries), stimulating steroidogenesis (for review, see [175]) and regulating the ovulatory process (for review, see [176]). Androgens and estrogens negatively feedback to the hypothalamus and pituitary to downregulate GnRH and gonadotropin secretion (for review, see [177,178]). The literature on gonadal steroid negative feedback comprises a number of papers detailing the mechanisms at the level of the hypothalamus and pituitary, but there is not a comprehensive review found of this to date.

Recent research calls into question the precision of the classic HPG nomenclature. For example, there are also GnRH and GnIH receptors in the gonads, which has additional implications for how these “neuropeptides” function at the level of the gonads (for review, see [179]). Additionally, estrogens are necessary for proper spermatogenesis and testicular function [180,181,182]. Referring to estrogen as the “female hormone” in scientific literature potentially postponed research on this important role for estrogens in testes. How we limit the questions we ask as researchers by using socially-constructed language in scientific nomenclature is evidenced by these few examples in the HPG axis.

The HPG axis is in constant interface and influence by factors such as social cues [183] and aging [178,184,185]. This introduction focuses on the interaction between melatonin and seasonality of the vertebrate reproductive axis. Specifically, melatonin and the HPG axis are reviewed here in post-pubertal mammals, birds, and other non-mammalian vertebrates. For a review of the HPG axis during puberty, see [185].

### 4.2. In Mammals

**Figure 5:**
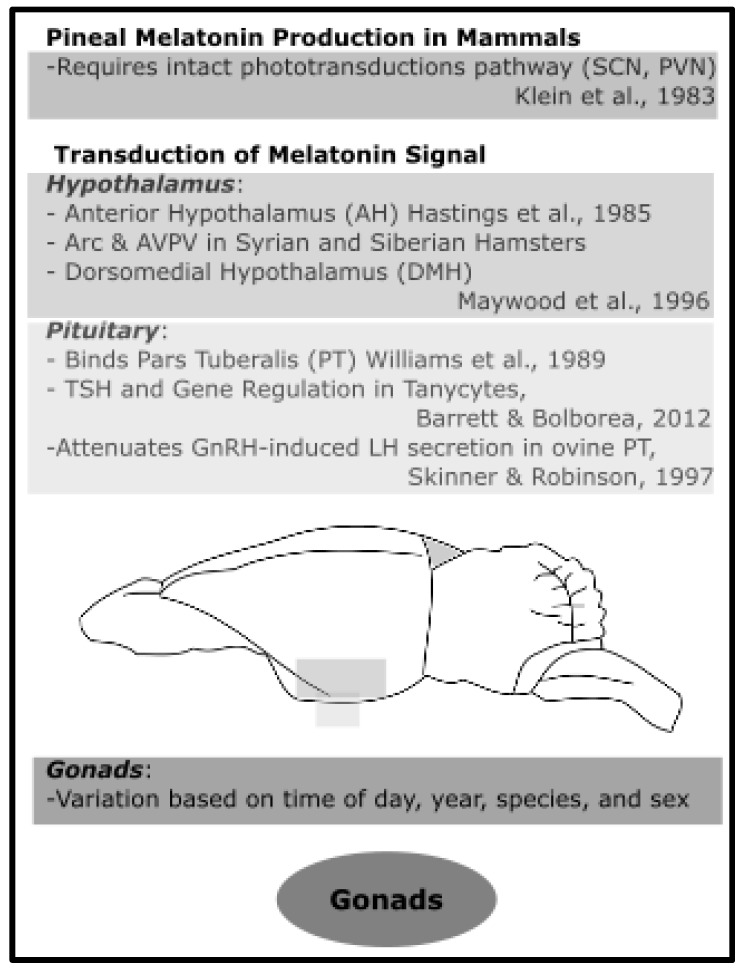
Summary of major findings on melatonin in the mammalian HPG axis.

The mammalian brain and the gonads contain melatonin-synthesizing sites (for review, see [123]). Given that pinealectomies prevent light-induced changes in the reproductive state of photoperiodic mammals, we may deduce that pineal-derived melatonin is necessary for maintaining reproductive physiology in seasonal, photoperiodic breeding mammals. Furthermore, there appears to be no compensatory mechanism for melatonin synthesized in other regions. This section focuses specifically on the effects of melatonin in mammalian hypothalamus, pituitary, and gonads in relation to seasonal reproduction (see Figure 5 for summary).

### 4.3. Melatonin in Mammalian Hypothalamus

The hypothalamus integrates environmental and physiological information to inform everything from satiety to osmoregulation to reproductive state. In previous sections of this introduction, the mammalian hypothalamus has been described in terms of its role as a conduit in the mammalian phototransduction pathway and as a melatonin binding site. Here, we focus on how the mammalian hypothalamus integrates photoperiodic and/or melatonergic signals to affect the reproductive condition of different breeding types of mammals (for an additional review on this topic, see [186]).

While evidence for immediate and direct effects of melatonin on deiodinase regulation of reproductive state is accumulating (see next section on Mammalian Pituitary), there is also research demonstrating direct effects of melatonin on mammalian reproductive neuropeptides in the hypothalamus. Melatonin affects gonadotropin-releasing hormone (GnRH) neuronal control via the stimulatory signal of Kisspeptin (*Kiss*) and inhibitory signal of RFRP (the mammalian homolog of gonadotropin inhibitory hormone, GnIH). For mammals, these neuropeptides are involved in the transduction of photoperiodic signals into gonadal responses (for review, see [186]). Specific hypothalamic nuclei in the phototransduction pathway also regulate pineal melatonin synthesis. The suprachiasmatic nucleus (SCN) and paraventricular nucleus (PVN) are hypothalamic nuclei that must be intact to regulate melatonin synthesis in mammals [187]. Lesions to the SCN or PVN remove the pineal melatonin signal [188]. However, appropriately timed melatonin injections still induce testicular regression in hamsters [188]. In this particular study, lesions that extended to neighboring hypothalamic nuclei (i.e., the anterior hypothalamus, AH) were associated with the variability observed in testicular response [188]. By using neurotoxic lesions that destroy neuronal cell bodies of the AH without affecting fibres passage or glia, leaving the non-sensitive neurons of the SCN and PVN intact, Hastings [189] determined cell bodies of the AH are necessary for photoperiodic regression of Syrian hamster testes during short-days. Damage to pericellular areas of the the AH, such as the POA, was not correlated to changes in testicular response associated with photoperiodic time measurement [189]. Intracranial application of melatonin suggested that melatonin acted on a site near the SCN but not the SCN directly [190]. Therefore, hypothalamic nuclei outside the phototransduction pathway were studied as critical sites of melatonin action influencing seasonal changes in mammalian reproductive state.

Other methodological approaches isolated the impact of melatonin on the mammalian hypothalamic network overall. In hypothalamic cell lines in rats, administration of melatonin downregulates *Kiss* and upregulates RFRP [191]. Beyond cell lines, specific mammalian hypothalamic nuclei that are not a part of the phototransduction pathway also influence reproductive physiology. In hamsters housed in photo-inhibitory short days, *Kiss* expression was down-regulated in the anteroventral periventricular nucleus (AVPV) of both Syrian and Siberian hamsters and the arcuate (Arc) nucleus of just the Syrian hamster. Peripheral administration of kisspeptin to short-day housed Syrian hamsters with regressed testes significantly increased testicular volume and testosterone secretion, and this response was blocked by GnRH receptor antagonist [192]. Melatonin injections downregulated *Kiss1* expression, and removal of the pineal prevented short-day inhibition of *Kiss1* in the Arc of the Syrian hamster hypothalamus (for review see [165,193]). From these studies, we find that melatonin affected *Kiss1* expression and kisspeptin mediated a reproductive signal via a pathway responsive to GnRH [192]. This complements findings suggesting that GT1-7 cells containing GnRH-secreting neurons expressed melatonin subtype receptors [194] and that melatonin receptor activation regulated GnRH gene expression in these GT1-7 cells [195]. These results have yet to be determined in vivo. It is difficult to ascertain the transferability of these findings from isolated cells to the whole animal. The underlying hypothalamic regulatory mechanisms vary for hamsters experiencing testicular regression, photostimulation of testicular growth, and spontaneous recrudescence of testes in extended photo-inhibitory conditions. The role for melatonin reflected by these different reproductive states will be contingent on photic conditions. Neither the subtleties of reproductive state nor the responsiveness to light (contingent on photoperiodic history) can be fully re-enacted in vitro.

The role for melatonin on the mammalian hypothalamus was studied in vivo by administering melatonin or changing in the lighting schedule. One study showed that chronic cannulae administration of melatonin into the medial hypothalamus of pinealectomized male Syrian hamsters maintained photostimulated testicular volume, and this was not observed for melatonin administered to the lateral hypothalamus, midbrain, or amygdala [190]. Furthermore, androgen receptors (AR) and melatonin receptors are co-localized in the dorsomedial hypothalamus (DMH) [196]. Testicular regression in Syrian hamsters, typically induced by extended melatonin infusions, were prevented with DMH lesions [196]. DMH ablation prevented photoperiod-induced testicular regression without affecting negative feedback on FSH in the pituitary in Syrian hamsters [197]. Furthermore, RFRP-ir and mRNA expression was significantly reduced in Syrian hamsters housed in short-days relative to long-days [198]. Testosterone administration does not appear to affect RFRP, suggesting a steroid-independent mechanism of RFRP regulation in the DMH [198]. *Rfrp* neuronal expression, typically associated with inhibition of the reproductive axis, was highly down-regulated in the DMH in male Syrian hamsters housed in short days [199]. Chronic administration of RFRP-3 led to testicular recrudescence and *Kiss* upregulation in the Arc nucleus in the male Syrian hamster, despite being housed in photo-inhibitory conditions [199]. RFRP-3 expression in the DMH is strongly inhibited by chronic infusion of melatonin in male Syrian hamsters [200], counter to what was observed in the rat hypothalamic cell line [191]. These findings show the importance of considering individual hypothalamic nuclei in addition to considering the hypothalamus as a whole. Both Syrian and Siberian hamsters show variation in *RFRP* mRNA resulting from changes in melatonin levels due to ablation of the pineal and exogenous replacement of melatonin, and this relationship is not observed in the non-photoperiodic Wistar rat [165].

There are sex differences in the regulation of reproductive neuropeptides in hamsters. More RFRP neurons were counted in female Syrian hamsters relative to males [201]. Female Syrian hamsters housed in short-days had downregulated RFRP in the AVPV, so there are sex differences in how RFRP is regulated in Syrian hamsters [201]. Although seasonal gene regulation was determined in male Siberian hamsters [202], seasonal gene regulation is likely to be different in females.

As for short-day breeding mammals, melatonin’s effect on reproductive state was studied mostly in sheep (for review, see [203]). The mechanism linking melatonin and kisspeptin in sheep is still not clear. The ovine premammilary nucleus binds melatonin [55]. However, melatonin receptor subtype MTNR1A was not found to be expressed in kisspeptin neurons but MTNR1A was found in the ovine pars tuberalis to regulate prolactin, which has indirect effects on kisspeptin [204]. MTNR1A polymorphisms showed no significant relationship to “out-of-season lambing” [205]. When melatonin binds particular subtype receptors sheep, subsequent molecular and physiological responses are yet to be distinguished. More research elucidates the kisspeptin/RFRP systems in sheep, which alternate in peak levels between reproductively active and quiescent cycles [206]. Long -days are associated with upregulation of RFRP in the ovine hypothalamus [207]. The isolation of melatonergic, steroid-independent effects of seasonal changes in *RFRP* expression has yet to be determined in sheep.

Although the mode of signal transduction between melatonin and the mammalian kisspeptin/RFRP system is neither fully elucidated nor conserved, there is clearly an upstream role for melatonin on neuropeptides in the mammalian hypothalamus that influence GnRH via RFRP and Kisspeptin. Future studies in mammals can consider how melatonin interacts with the circadian pulsatility of the GnRH system (for review, see [208]). Furthermore, the positive and negative feedback of estradiol on hypothalamic neuropeptides would need to be integrated into future studies on the relationship between melatonin and female reproductive state (for review, see [209]). The pars tuberalis of the pituitary, as a conserved binding site for melatonin across different breeding types of mammals, shall be discussed in the subsequent section.

### 4.4. Melatonin in Mammalian Pituitary

Melatonin binds the pituitary of rat [210], Syrian hamster [52], and in rhesus monkeys (*Macaca mulatta*) [211]. Melatonin binding was observed only in one out of the eight human pituitaries [211]. As discussed in the section on Melatonin Binding, the pars tuberalis is a conserved melatonin binding site in mammals (for review, see [48]). There is no single cohesive narrative to how melatonin acts in the mammalian pituitary to affect reproduction, so the literature reviewed here provides a heterogenous perspective of the physiological effects of melatonin binding in the mammalian pituitary.

Most research on melatonin action in the pituitary was conducted in rats. Nanomolar concentrations of melatonin, but not micromolar, administered in vitro to anterior pituitary harvested from neonatal rats suppress LH/FSH release induced by luteinizing-hormone-releasing-hormone (LHRH) [212]. In other studies, melatonin prevented LHRH-induced cAMP and cGMP accumulation [213] and GnRH-induced intracellular free Ca^2+^ and depolarization of the plasma membrane [214] from cultured neonatal rat anterior pituitary. However, the pars distalis of the fetal rat has a higher responsiveness to melatonin that declines with development into adulthood [215], so these subcellular changes resulting from melatonin signalling in cultured fetal rat anterior pituitary is not ontologically analogous to melatonin action in the adult pituitary.

Melatonin action at the level of the pituitary of small mammals that are more photoperiodic than rats is also evident. In Syrian hamsters, a photoperiodic rodent, melatonin binds the pars tuberalis [52]. Melatonin binding in the Syrian hamster changes based on photoperiod in the median eminence and in the anterior pituitary [213]. The signal transduced by melatonin binding in the pars tuberalis (PT) of the pituitary includes regulation of thyroid-stimulating hormone (TSH), which in turn affects gene regulation in tanycytes, cells lining the third ventricle of the hypothalamus (for review, see [216]). The enzymes that activate or deactivate thyroid hormones, deiodinases *Dio2* and *Dio3*, have been a focus of how melatonin mediates reproductive responsiveness in hamsters. Dio3 inactivates T3 and its prohormone, thyroxine (T4), while Dio2 converts T4 into its active form T3. Triiodothyronine (T3) injections stimulate testicular growth and modulate neuropeptide synthesis to activate reproductive physiology in photorefractory Siberian hamsters [217]. While exogenous administration of thyroid hormones appears to override the impact of photoperiod on reproductive state, exogenous melatonin also can affect the Dio2/Dio3 system. Melatonin injections increase *Dio3* expressions levels in juvenile Siberian hamsters, implicating a melatonergic effect in peripubertal maturation [218]. Photostimulated adult Syrian hamsters that were injected with melatonin for one week showed levels of *Dio2* mRNA comparable to what is observed in hamsters kept in short days [219]. However, the effects of melatonin injections on *Dio2*/*Dio3* expression are inconsistent based on the time of day of the injection and the strain of mice used [220]. A single melatonin injection administered in the late afternoon alters the temporal dynamics of *Dio2* expression the subsequent day in Syrian hamsters [221]. Furthermore, melatonin injections used to simulate short-days and terminate breeding differentially affects Dio2/TSHβ relative to long-day induction, or photostimulation, of this pathway [222]. Melatonin injections and light pulses have differing effects on *Dio2* in Siberian hamsters. *Dio2* expression decreased with melatonin injections but did not change with light pulses [223], suggesting that melatonin serves as an intermediate between changes in lighting condition and *Dio2* regulation. In male Syrian hamsters, the rapid induction of TSHβ expression in the pars tuberalis, determined by in situ hybridization, following photostimulation is a strong indicator of the role of the pituitary in seasonality of the HPG axis [222]. However, melatonin injections simulating short-days in this long-day breeder did not synchronously alter Dio2/TSHβ mRNA expression, suggesting another mechanism is involved in the termination of breeding [222]. Additionally, in male viscacha (*Lagostomus maximus*), chronic melatonin administration (twice daily s.c. injections for 9 weeks) decreased the size of LH and FSHβ cells [224]. Chronic exposure (16 h) to melatonin in mediobasal hypothalamic explants including the pars tuberalis showed lower melatonin binding in the pars tuberalis in mink (*Mustela vison*) at all times of the year the tissue was harvested [225]. For further reading on the molecular mechanism of Dio2 see [226].

Larger seasonally breeding mammals provide certain advantages over small mammals in understanding the temporal dynamics and physiological nuances of melatonergic effects on pituitary function. Larger mammals can have more frequent blood sampling and offer more visibly accessible anatomy for intricate surgical procedures. One example of the value of regular blood sampling in ascertaining temporal dynamics is seen in a study conducted in reproductively quiescent dairy goat (*Capra*). From this study, the number of LH pulses were unaffected by lighting condition and melatonin treatment, but basal levels of LH increased with melatonin treatment [227]. The temporal scale of blood sampling needed to distinguish LH pulsatility from estrous rhythms was more easily enabled by using a larger mammal. The indirect effects of melatonin on the pituitary via hypothalamic neuropeptides are difficult to distinguish from the direct effects of melatonin binding the pituitary without damaging other necessary physiological systems. One model used to distinguish these effects, enabled by the size of the animal, is pituitary disconnected rams (PDR). The hypophyseal portal system is compromised in PDR. Melatonin implants in PDR have an effect on prolactin secretion that is comparable to control groups, suggesting that in this model, melatonin has a direct effect on the pituitary the functions independently from hypothalamic input [228]. As mentioned before, the mode of melatonin administration tend to vary across mammalian pituitary studies (in these examples, implant versus injection), and the results of these experiments can not be directly comparable in determining a conserved mode of action for melatonin on reproductive pituitary function across mammals. Lastly, the ovine pituitary itself is large enough to separate tissue fragments to study the effects of melatonin on the pars distalis and the pars tuberalis in vitro [229]. Administering melatonin to cultured ovine pars tuberalis, but not the pars distalis, attenuated the GnRH-induced secretion of LH [229], revealing a functional role for the high density of melatonin receptors previously found in the pars tuberalis of sheep.

The role for melatonin binding specifically in the pars tuberalis and its effect on local gene regulation was studied extensively. It is impossible to isolate metabolic effects from reproductive state, especially since body weight and reproductive success are seasonally regulated by melatonergic pathways in photoperiodic breeding mammals (for review, see [216]). The question remains about the role of melatonin in the human pituitary. In healthy adult males, melatonin administration did not correspond to changes in LH, FSH, or testosterone (*n* = 5 males over one night in [230]; *n* = 6 males over 3 nights in [231]). However, results from pituitary gonadotropin research with healthy adult females show a stronger relationship between melatonin and reproductive state. A significant effect of season on melatonin and LH concentrations was observed, regardless of menstrual state (*n* = 11 females providing serum and urine samples and *n* = 21 providing serum samples for anterior pituitary and ovarian hormones in [232]; *n* = 12 females, samples collected various days of menstrual cycle in summer and winter in [233]). Sex differences were previously observed in reproductive responses to stress (for review, see [234]). As extensively reviewed by [235], the sex bias in animal research can learn from these differences in humans. The effects of melatonin on pituitary reproductive function can not be extrapolated across sexes, and consideration of sex differences extends beyond the gonads.

### 4.5. Melatonin in Mammalian Gonads

Research related to sexual dimorphism of melatonergic effects on reproductive physiology tends to focus on the gonads, despite research showing that there is more than what meets the gonads (testes/ovaries) when it comes to sex differences in animal research [235]. Extensive reviews have been conducted on melatonin in seminal plasma [236], on oocyte competence and blastocyst development [237], on in vitro fertilization and embryo transfer success [238,239,240,241,242,243], on the ovary [113], and on male reproductive health overall [238,244]. However, parsing out the effects of melatonin acting as a hormone from other confounding factors (e.g., other products of melatonin biosynthetic pathway, melatonin synthesizing-enzymes, melatonin as an antioxidant/free-radical scavenger, etc.) is a challenging undertaking. In several studies, the direct endocrinological relevance of melatonin in the gonads is left to speculation. Since AANAT and ASMT show highest activity in the interstitial cells of rat testes [121], and early research shows melatonin and serotonin can inhibit key enzymes in androgen steroidogenesis in vitro [120], melatonin and the products of its biosynthetic pathway (see Figure 1) can also affect steroidogenesis in a manner that cannot be isolated through the methodologies implemented in these studies. The foundation of research on melatonin in reproductive organs provides key insights into the function of melatonin on reproductive state in seasonal reproductive breeding mammals.

Melatonin has varying effects on testicular physiology. For instance, sperm motility in humans seems to be unaffected by melatonin [245], inhibited by high concentrations of melatonin in Wistar rats [246] and hyperactivated in Syrian hamster [247]. These varying effects of melatonin on sperm motility might be due to time of day the tissue was harvested, photoperiodicity of these particular mammals, or even melatonin-receptor expression in spermatozoa. Melatonin implants during the non-reproductive season (spring) in Rasa Aragonesa rams (short-day breeders) increase seminal plasma testosterone concentrations after four weeks and 17β-estradiol after eight weeks [248]. From this, we can predict that for seasonally-breeding males that respond to melatonin to time reproduction, melatonin administration can override other endogenous circannual rhythms to activate the mammalian reproductive axis. Fifteen weeks of melatonin administration in the breeding season (winter) compared to the non-breeding season (spring) significantly increased plasma testosterone concentrations in both winter and spring in Chios rams, and this increase was higher in winter [249]. In Syrian hamster (long day breeders), testes collected at different reproductive states (photostimulated and photorefractory) showed melatonin concentration is significantly higher in the testes of hamsters kept on short days (photorefractory) relative to their long days (photostimulated) counterparts [250]. The concentration of melatonin in the testes correlated with the nanomolarity of *N*-acetyltryptamine formed, which was used as an indicator of AANAT activity [250]. In addition to the implication of seasonal changes in testicular melatonin synthesis, Mukherjee and Haldar also found MT1 receptor was significantly higher in testes collected from the short-day group [250]. It is worth noting that the time of day when the testes were collected was not specified in this study [250], so we are not able to assume the testes in different groups were collected at the same time. Across different breeding types, *MT1* and *MT2* are expressed in the spermatozoa of long-day breeders, short-day breeders, and non-seasonal breeders [118], so melatonin can affect sperm in a receptor-mediated fashion. This is supported by the finding that the melatonin receptor antagonist, luzindole, inhibits melatonin-induced hyperactivation of sperm motility in Syrian hamsters [247].

There is also evidence for melatonergic effects on ovarian physiology. Melatonin administered with human chorionic gonadotropin (hCG) decreased progesterone and estradiol production in preovulatory follicles in adult cyclic female hamsters [251]. Melatonin concentrations peaked in the hamster ovary in the middle of the scotophase, as observed in serum and in the pineal gland, suggesting melatonin in the ovary might inform this cyclicity [251]. In early preantral follicles harvested from C57BL × CBAca mice cultured with varying concentrations of melatonin, androstenedione and progesterone concentrations increased with 100 µM melatonin, but any concentration higher was toxic or negatively influenced oocyte maturation while lower concentrations had no effect [252]. In CD-1 mice, with stimulated follicle growth by pregnant mare serum gonadotropin (PMSG) and triggered ovulation by hCG, ovarian fluid showed increased concentrations of melatonin as well as *MT1* and *SNAT* (AANAT) expression in cumulus cells [253]. Additionally, melatonin administration increased progesterone and rates of successful implantation [253]. Similar findings of melatonin receptor and enzyme expression are observed in bovine cumulus-oocyte complexes [254]. Although these studies show melatonin-receptor expression alongside ovarian physiological changes, the lack of a melatonin-receptor antagonist means we can not distinguish with certainty the antioxidant effects of melatonin from receptor-mediated signal transduction.

The seasonal patterns of intra-gonadal melatonin synthesis and binding have yet to be studied. Measurements of melatonin from seminal fluid does not exclude extra-gonadal sources of melatonin (e.g., exogenously administered melatonin, see [248]), and the amphiphilic properties of melatonin allow diffusion across the blood-testis barrier. Furthermore, several studies do not not use receptor antagonists to distinguish melatonin receptor-mediated changes from actions of melatonin as an antioxidant. Seasonal variations of melatonin synthesis and secretion are worth considering.

### 4.6. In Birds

The effects of melatonin in timing reproduction in birds are not consistent across species or seasons. Synchronizing egg laying and care for fledglings with adequate environmental resources is necessary in a vertebrate class for which storing fat and lactating could compromise mobility. Seasonal changes in the avian HPG axis were reviewed focusing on the role of thyroid hormone [255]. Another review that investigated the role of melatonin in non-mammalian vertebrate reproduction by closely considering the methodology of research on melatonin in avian reproduction [256]. The methodologies addressed in [255] included studies that administered of melatonin [257,258,259,260,261,262,263,264] or its antisera [265] in birds. We can attribute the inconsistent effects of melatonin on avian reproduction to methodological variation of these administration studies.

Additionally, the methodology of most of the studies investigating the effects of melatonin on avian reproduction conducted bilateral enucleation and/or pinealectomies [256]. However, melatonin rhythms are still detectable in pinealectomized and bilaterally enucleated male Japanese quail (*Coturnix japonica*) at 13% of the concentration measured in intact controls [12]. This might be due to post-pinealectomy compensatory mechanisms that develop over time, as observed in other birds. Although plasma melatonin is undetectable in pinealectomized White leghorn cockerels (*Gallus gallus domesticus*) [266], melatonin rhythms are maintained post-pinealectomy in the eye and Harderian gland, with local melatonin concentrations peaking in amplitude during the dark phase [267]. In pigeons (*Columba livia*), extra-pineal melatonin was found in the hypothalamus, eyes, Harderian gland, and duodenum [268], and allowing two weeks recovery post-pinealectomy showed increases in detectable levels of circulating melatonin up to 64% of amount quantified in sham-operated controls [269]. Unlike photoperiodic mammals, American tree sparrows (*Spizella arborea*), even after pinealectomy and bilateral enucleation, experienced testicular growth when photostimulated [11]. It is important to consider that these birds have extra-pineal, extra-ocular sources of rhythmic melatonin synthesis not found in mammals (a comparative discussion of melatonin in relation to thyroid hormone is presented in [224]).

**Figure 6:**
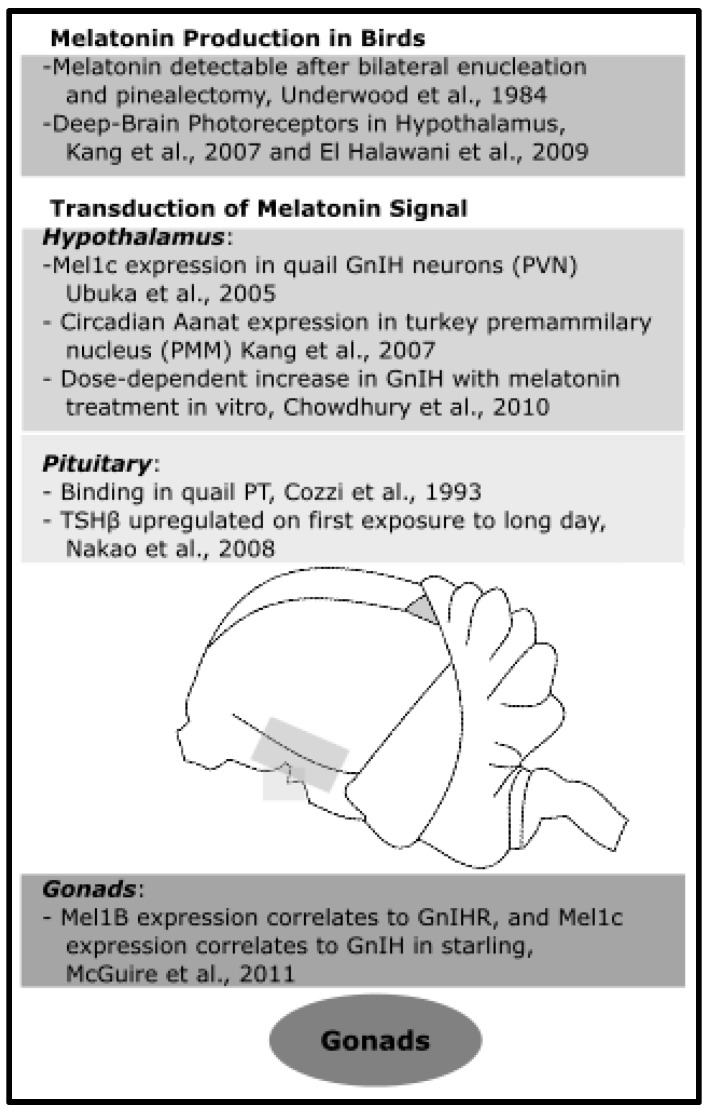
Summary of major findings on melatonin in the avian HPG axis.

Furthermore, some birds, unlike mammals, have photoreceptors in the pineal situated beneath a translucent skull and deep brain photoreceptors localized to the hypothalamus (see Phototransduction Pathways in Mammals and Birds). The role of melatonin in circadian rhythms, seasonal immune function, and neuroplasticity in the song control network was reviewed previously [270,271]. Here, the mechanisms underlying how melatonin interacts with the hypothalamus, pituitary, and gonads to affect avian reproduction are reviewed in different breeding types of birds (for reviews on the effects of photoperiod on avian reproductive state, which includes but is not limited to melatonergic effects, see [272,273]). See Figure 6 for summary of major findings.

### 4.7. Melatonin in Avian Hypothalamus

The relationship between reproductive neuropeptides and photoperiodic time measurement (PTM) has been extensively reviewed [171,274]. The role of hypothalamic melatonin can be partially understood through an exploration of PTM. The avian mediobasal hypothalamus (MBH) includes the infundibular nucleus, inferior hypothalamic nucleus, and median eminence, and numerous studies indicate the MBH is necessary for PTM to induce gonadal growth or regression to correspond with changes in photoperiod (MBH lesions [275,276], MBH electrical stimulation [277], and photo-induction of immediate early gene c-Fos expression [278,279]). Although i.v. injections of anti-melatonin serum before lights-out in Japanese quail enabled testicular development during short-days [265], we can not isolate which part(s) of the HPG axis the anti-melatonin serum bound. Whether anti-melatonin serum acted at the level of the hypothalamus, pituitary, the gonads, or some combination of these classical target tissues to enable photostimulation in quails housed in short-days is worth investigation in future studies. It is worth noting that these studies use only male Japanese quail, and other studies have indicated there are interspecies and sex differences [280]. To generalize from findings in male Japanese quail to birds of all sexes and breeding types can not depict the most accurate representation of the role of melatonin in the avian reproductive axis.

More species have been integrated in research using 2-[^125^I]iodomelatonin binding to identify melatonin receptors in the avian brain, which labels sites where the affinity and density are high enough to cross a critical threshold (reviewed in the section on Melatonin Binding in this Introduction). Previous 2-[^125^I]iodomelatonin studies in birds identified binding in the hypothalamus to be restricted to the visual pathway, specifically to the visual suprachiasmatic nucleus, or vSCN (in chicks [124], in house sparrow *Passer domesticus* [281], in budgerigar *Melopsittacus undulatus*, cockatiel *Nymphicus hollandicus*, northern cardinal *Cardinalis cardinalis*, *Melospiza melodia*, European starling *Sturnus vulgaris*, chicken *Gallus gallus*, common pheasant *Phasianus colchicus*, helmeted guineafowl *Numida meleagris*, Virginia or bobwhite quail *Colinus virginianus*, rock pigeon *Columba livia*, ring dove *Streptopelia risoria*, mourning dove *Zenaida macroura*, and mallard duck *Anas platyrhynchos* [63]). However, sequencing of specific subtype receptors increased the resolution to detect melatonin receptors to individual neurons. When the relationship between melatonin and GnIH mRNA and peptide in the diencephalon of Japanese quail was found to be dose-dependent, the mode of melatonergic action on GnIH in the paraventricular nucleus (PVN) was investigated [129]. In addition to *Mel1c* expression in the PVN, in situ hybridization co-localized this melatonin subtype receptor with immunolabeled GnIH cell bodies [129]. Since the melatonin receptor autoradiogram showed relatively low binding in the PVN compared to neural regions with high binding [129], it seems that multiple methods are necessary for identifying melatonin binding and subsequent action in the avian hypothalamus.

In addition to studies on circadian clock gene expression in the MBH and SCN [282] , along with differential subtractive hybridization analysis in quail [282], a photoperiodic model in quail appears to include thyroid hormone synthesizing-enzyme *Dio2* similarly to photoperiodic breeding mammals (see section on Melatonin Signalling In Mammals). In European starlings, *Dio2* varied seasonally but did not correspond to testicular volume or GnRH regulation in males [283], and in females, first evidence was presented for *Dio2* expression to be regulated by social cues (i.e., the presence of a mate) [284]. The direct relationship, if any, between melatonin and Dio2 in birds remains unclear to date. Melatonin in birds is mostly reviewed in relationship to its daily rhythm, dissociated from the seasonal photo-neuroendocrine reproductive axis [285], because of results of melatonin administration studies in birds, which are more inconsistent than administration studies in photoperiodic breeding mammals.

Recent studies uncover the potential for melatonergic action in the avian hypothalamus. In addition to the discovery of *Mel1c* expression in GnIH neurons of the quail PVN previously described [129], circadian patterns of melatonin and AANAT were characterized in the turkey (*Meleagris gallopavo*) premammilary nucleus (PMM) with an inverse relationship with the co-localized expression of dopamine and tyrosine hydroxylase [13,14]. The photoperiodic condition and corresponding reproductive state of the turkey also affected these expression patterns and associated with changes in *GnRH-I* mRNA expression [14]. Additionally, changes in mRNA expression of the first enzyme in melatonin synthesis (*TPH1*) corresponded to expression of a photoreceptor gene (melanopsin, or *OPN4*), both of which decreased in a time-dependent manner in the PMM of photosensitive female turkey hens exposed to light [286], indicating a relationship between deep-brain photoreception and melatonin synthesis.

In another study, hypothalamic explants (including the PVN and GnIH projections to the median eminence) from adult male Japanese quail were cultured to determine the effect of melatonin on GnIH release [287]. *GnIH* mRNA and GnIH peptide dose-dependently increased in tissue cultured with higher melatonin concentrations [287]. Tissue also was collected at different times, and GnIH peptide release peaked in the dark period in coincidence with melatonin concentration in the diencephalon and inversely related to LH concentration in plasma [287]. Furthermore, quails that were housed in short days for 3 weeks had significantly higher *GnIH* mRNA and peptide released in the culture media and significantly lower concentrations of LH in plasma and testicular volume [287], correlating observations in cultured quail hypothalami with the HPG of quail in different reproductive states. The remaining detectable melatonin concentrations in the diencephalon were hypothesized to be derived from pineal and ocular sources, since their previous study showed that removal of these tissues significantly decreased melatonin concentrations in the diencephalon [129]. However, there was still melatonin detectable in the diencephalon of pinealectomized and bilaterally enucleated quail [129].

In summary, the direct action of melatonin on GnIH neurons is key to understanding the physiological mechanism by which photoperiodic information is chemically transduced to affect GnRH-I and GnRH-II (in European starlings [288], in Indian weaver bird, *Ploceus philippinus* [289], reviewed in [290]). The hypothalamus hosts GnIH neurons, which express Mel1c receptors [129], and also deep-brain photoreceptors that can regulate local melatonin synthesis [288]. Furthermore, given the distribution of GnIH and its receptors throughout the avian reproductive system (for review see [291]), melatonin might also regulate gonadal development and regression via GnIH in these other sites. Melatonergic action in the avian pituitary and gonads discussed in the subsequent sections.

### 4.8. Melatonin in Avian Pituitary

The avian pituitary hosts a wide variety of hormones broadly related to reproduction. There are several studies investigating gonadal or behavioral response to changes in prolactin (for review, see [292,293]), adrenocorticotropic hormone (ACTH) [294,295,296], arginine vasotocin [297,298], and mesotocin, the avian homologue of oxytocin [297,299]. The seasonal fluctuations of these pituitary hormones directly precede, coincide, or are inversely related to gonadal steroids. These hormones have been studied in connection to the onset of broodiness [300,301], yolking [302], nesting & incubation [301,303], and cessation of laying [300]. This section will focus on the relationship between melatonin and pituitary hormones that are most strongly connected to avian reproductive activation include gonadotropins, LH/FSH, and thyrotropin, TSH.

Melatonergic actions at the level of the avian pituitary can regulate reproductive gonadotropins indirectly via GnIH as described in the previous section. Regarding the potential for direct action, Although mammals share a conserved melatonin binding site in the pars tuberalis, no 2-[^125^I]iodomelatonin binding has been observed in the median eminence/pars tuberalis of avian species [62]. However, upon closer investigation, we find that Japanese quail showed binding in the hypophyseal pars tuberalis [125]. Binding was not detectable in the adenohypophysis, neurohypophysis, nor the median eminence in house sparrows [281] nor was pituitary binding even mentioned for an autoradiographic study in the chicken brain [304]. However, expression of Mel1c subtype receptor was found in the chick pars tuberalis [305].

In the adult male Lal munia (*Estrilda amandava*), melatonin was injected with four different dose regimes of melatonin over thirty days (either mid-day or mid-night, low or high dose, and increasing or decreasing dosage over time), and seasonal gonadal growth was inhibited regardless of the time of day melatonin was administered [306]. Interestingly, melatonin injections increased colloid in the follicular lumen of the thyroid, and more thyroidal follicular cells become squamous, or inactive. As a proxy for the effects of melatonin on LH, feather and beak coloration were assessed. Melatonin injected birds lacked LH-induced changes in plumage pigmentation observed in controls. Circulating levels of FSH were not quantified, but it was concluded, since melatonin did not affect LH-induced changes in beak coloration, that melatonin might inhibit FSH as explanation for inhibition of testicular growth observed in the melatonin treated group. This can be due to localized changes in LH-receptor. Neither circulating LH or its receptor, however, were quantified in this study [306], so we can not conclude with certainty how melatonin affected LH synthesis or secretion from the anterior pituitary in this species.

In castrated White Leghorn roosters, melatonin injections at different concentration and administered for different frequencies or over extended periods of time consistently supported the hypothesis that melatonin dose-dependently reduces circulating LH levels [307]. Neither the mechanism between the photoperiodic signal to melatonin nor from melatonin to the LH signal are fully understood.

One possible mechanism for photoperiodic regulation of the melatonin signal on the avian pars tuberalis is via clock gene regulation (for a review on avian clock genes, see [308,309]). There is a rhythmic expression of *Cry1* and *Per2* in the pars tuberalis of Japanese quail, and this rhythm delays under longer photoperiods [310]. Furthermore, a light pulse administered during the dark period can induce *Cry1* expression [310]. Lastly, the role of clock genes in regulating the effects of melatonin on the avian pituitary is shadowed by research on thyrotrophin of the pars tuberalis. A seminal study was conducted in male Japanese quail that were 8 weeks old investigating photoinduced changes in gene expression upon exposure to the first long day [285]. Thyrotrophin (*TSH*) β-subunit expression was significantly upregulated in the pars tuberalis 14 h after lights-on of the first long day, and this was followed 4 h later by increased *DIO2* expression [285]. Furthermore, administration of TSH to quail housed in short days stimulated testicular development, and *DIO2* expression appears to be regulated in part through a signalling pathway involving TSH receptor-cyclic AMP [285]. This study provides compelling evidence for thyrotrophic-mediated signalling between photostimulation and testicular development in quail. However, there are notable details of the study that limit the potential ecological relevance of these findings. Quail were housed in square-wave pattern (on-off) lighting and were switched directly from short days (6L:18D) to an extremely long-day (20L:4D). The missing transitory cues provided by natural simulation of dawn and dusk, including color [311] and irradiance [312,313], in addition to the dramatic shift in daylength that might only be experienced by migratory birds in the wild, limits the ecological generalizability of this study. Additionally, quail were selectively bred and are considered to be weakly photoperiodic, so the implications for other breeding types remain unclear.

The first study to consider the ecological relevance of early gene expression in photoperiodic time measurement was conducted in wild-caught great tits (*Parus major*) [314]. Male great tits were collected in Sweden (57°42′ N) and Germany (47°43′ N). Upon exposure to one long day, the great tit population from Sweden significantly changed hypothalamic gene expression while the population from Germany did not [314]. Despite only one population showing changes in hypothalamic gene expression, both populations significantly increase gonadotropin secretion and synthesis [314]. It is plausible that the pituitary in this species is differentially responding to photoperiodic cues beyond changes in hypothalamic gene expression, and it is also worthwhile to consider that hypothalamic early gene expression is not conserved mechanism underlying photoperiodic regulation of the avian HPG axis. Another aspect that will be investigated here is the relationship between melatonin and the proxy for reproductive state in photoperiodic breeding birds: the gonads.

### 4.9. Melatonin in Avian Gonads

In the Melatonin Binding section of this introduction, melatonin binds in the testes and ovaries of chicks, ducks, and quail, but not in mammals [117,315]. Only recently could we begin to hypothesize the functional role for melatonin within the avian gonad in its effect on reproductive state and timing. Avian gonads express reproductive neuropeptides (such as GnRH and GnIH) and their receptors (for review, see [179]). European starling testes express melatonin receptor subtypes Mel1B and Mel1C [132] and also express melatonin synthesizing enzymes (unpublished data). The potential for autocrine or paracrine regulation of gonadal melatonin on its own synthesis or the synthesis of its receptors can be corroborated by evidence of melatonin regulating itself in this fashion in the mammalian brain [102]. Here, the focus is on how melatonin interacts with the avian HPG axis at the level of the gonads.

The reproductive state the testes were cultured in also affected the effect of melatonin on testosterone production. Before the breeding season, melatonin upregulated *GnIH* mRNA in the testes, and GnIH and melatonin combined led to a significant reduction in testosterone secretion of starling testes in vitro as well [133]. While GnIHR expression increased with photoperiod, GnIH expression appeared to correspond with testicular volume, for GnIH significantly higher in photosensitive (February) and photorefractory (June) birds than in photostimulated (April) birds [133]. Additionally, there was a significant correlation between *Mel1B* and *GnIHR* as well as between *Mel1C* and *GnIH* [133]. Because *Mel1C* expression significantly decreased in testes treated with LH/FSH, there appears to be a causal relationship between reproductive state and this melatonin subtype receptor expression in starling testes [133].

In avian ovaries and follicles, melatonin receptors have been sequenced varied stages of follicular development [132] and egg laying [316]. Melatonin implantation (10 mg) to chickens 300–470 days of age increased egg-laying rate [316]. Chickens supplemented with melatonin had no significant difference relative to controls with LH-receptor, FSH-receptor, estradiol receptor alpha (ERα), nor MT2 receptor expression in the ovaries [316]. However, chickens supplemented with melatonin implants did show significantly higher serum estradiol-17β concentrations and ovarian MT2 expression with significantly lower ovarian GnIH-receptor expression [316]. It is unclear what ecologically relevant phenomenon a tonic-release melatonin implant is simulating. However, these findings illustrate the potential for melatonin to bind the ovary and correspond to significant changes in reproductive physiology and fitness in chickens.

The effects of melatonin on egg laying were tested in free-living great tits (*Parus major*) [317]. Without affecting body mass, clutch size, or the daily onset of activity, silastic implants containing melatonin led to a significant delay in laying of the first clutch in female great tits in the wild [317]. The rate of egg laying, however, was not determined in this study, so we cannot make broader comparisons yet of the effects of melatonin on avian egg laying in general. In Indian jungle bush quail (*Perdicula asiatica*) housed in open air aviaries, an inverse relationship in the mass of the pineal and the mass of the ovary over the course of a year corresponded to seasonal changes in circulating melatonin [318]. Furthermore, peak adrenal activity corresponded to peak gonadal activity, which occurred in the season when plasma melatonin concentrations were lowest [319]. Whether these systems are independently affected by photoperiodic cues or responding to indirect endogenous signals is yet to be determined.

The relationship between melatonin, reproduction, and other cues cannot be entirely extrapolated. Given that there is seasonal responsiveness to cues of stress affecting testosterone and estradiol production in cultured testes and ovaries from starlings [320], then we can predict that melatonin also has a seasonal efficacy. This hypothesis is supported by findings showing seasonal regulation of melatonin subtype receptors in the testes of birds [133,134]. The seasonal pattern of peripheral melatonin coincides with photoperiod and is inversely related to circulating testosterone levels even in male Indian tropical bird (*Perdicula asiatica*) [321]. These findings would have extensive implications for research on melatonin and avian reproduction. If research showing melatonin has no significant effect on gonadal development were administering melatonin in a time or fashion when gonadal responsiveness/sensitivity was low [259,262,264,322], then the lack of a response would not be attributed to the generalized role (or lack thereof) for melatonin in avian reproductive development but rather a consequence of improper timing of administration.

### 4.10. Summary

There is variable evidence around the extent to which melatonin influences reproductive state in different breeding types of birds. Therefore, multiple non-photoperiodic cues should be considered in conjunction with seasonal changes in melatonin for a comprehensive picture of the avian HPG axis. These cues can include steroid dependent changes in sexual behavior or hypothalamic activation in response to social cues. In the non-photoperiodic breeding zebra finch (*Taeniopygia guttata*), castrated males were supplemented with androgenic and estrogenic compounds restored courting and mounting sexual behaviors [323]. Females solicited castrated males supplemented with estrogenic compounds at a significantly higher rate, even if the male was not exhibiting courtship behavior [323]. Additionally, social cues such as the presentation of female zebra finches to males did not significantly affect hypothalamic expression of *Dio2*, *Dio3*, *GnRH*, nor *GnIH*, but an immediate early gene (early growth response protein-1, or EGR1) did significantly increase relative to isolated males [324]. However, these studies focus on the male response to the presentation of a female and do not investigate the active role of the female. Our terminology can limit us to some extent because female songbirds “solicit” and male songbirds “court”, depicting historically constructed, anthropomorphic projections that limit the questions we ask and how we answer them scientifically. We need to reconsider how we frame sexual behavior in animal research, so we do not limit ourselves with our terminology. There are countless more questions to be addressed in the role of melatonin in sexual behavior and accompanying physiological changes in future studies.

Overall, there is more evidence supporting the role of melatonin in seasonal changes in song regulation [325], but melatonin does not have a consistent role in affecting avian gonadal state [326]. Research on melatonin in avian gonads might be limited because injections, oral administration, and subcutaneous implants containing a range of melatonin concentrations have widely variable results that are incomparable to the effect of melatonin observed in photoperiodic breeding mammals. This has led to the dismissal of melatonin as a necessary and sufficient contributor to seasonal changes in the avian HPG overall. However, from the studies listed above, we cannot dismiss that there is still a great deal to understand if and how the photoperiodic signal is relayed to the gonads in different breeding types of birds if it is not primarily by melatonin. This signal is likely mediated through indirect changes in the hypothalamus and pituitary as well as direct autocrine/paracrine regulation within the gonad itself.

### 4.11. In Other Vertebrates

There is an impressive range of ways in which melatonin integrates (or not) with reproductive signals in reptiles, amphibia, bony fishes, and cyclostomes [256], and many of these studies were conducted in controlled lab environments. The relationship between melatonin and seasonal reproduction might vary based on the anatomy and photoreceptivity of the pineal [327,328]. The structure of pinealocytes in snakes resembles those of mammals, lacking photosensory anatomical features [328,329]. Hagfish (*Eptatretus burgeri*) appear to lack a pineal complex [330,331]. A pineal complex has not been identified in crocodile, and circulating melatonin rhythms are absent in free-living crocodiles (*Crocodylus johnstoni*) but are present in captive populations exposed to natural light and temperature cycles [332], suggesting an extra-pineal source of circadian melatonin in this species. Additionally, the presence of a melatonin-synthesizing pineal gland in a given class of vertebrates does not imply that it has been studied in the context of seasonal reproduction. Lamprey pineals maintain diurnal patterns of melatonin synthesis in vitro (in *Petromyzon marinus* [144] and in *Lampetra japonica* [145]), but melatonin was not mentioned in recent reviews on the lamprey HPG axis [333,334]. This section will focus on research that has investigated the connection between melatonin and reproductive state in seasonal breeders across non-mammalian and non-avian vertebrate classes.

The daily cycle of melatonin and the effects of temperature on melatonin synthesis in poikilotherms were previously reviewed [256]. The majority of studies found investigating the effects of melatonin on reproduction in reptiles have been conducted in lizards. In the female Carolina anole lizard (*Anolis carolinensis*), pinealectomies at different times of the year have differential effects on ovarian status [335]. The gonadosomatic index (GSI) and the number of follicles yolked were significantly higher in the pinealectomized (Px) group administered saline relative to the Px group administered melatonin (daily s.c. injections of 10 µg) and all other control groups in January [335]. In June, the number of yolked follicles was significantly higher in the Px group administered saline, and in November, the ovarian GSI was significantly larger in the Px group administered saline, suggesting that the way in which pineal-derived melatonin affects ovarian status varies seasonally in this species [335]. It is important to note these animals were kept in constant 6L:14D lighting conditions [335], so the differential effects of melatonin vs. saline injections were not photoperiodically driven. Additionally, the effects of photoperiod on reproductive state in Px individuals were studied in male Carolina anole lizards [336]. Firstly, pinealectomy only affected reproductive state of the male lizard when the surgery was conducted in December, accelerating testicular response to photostimulation (14L:10D) relative to sham-operated individuals [336]. Secondly, also housed in the 14L:10D lighting condition in December, lizards of the Px group with blank silastic implants had significantly higher testicular volume compared to the Px group with implants continuously releasing melatonin [336]. The effects of pinealectomy and subsequent melatonin administration are comparable between male and female Carolina anole lizards [335,336]. In male Indian garden lizards (*Calotes versicolor*), if the pinealectomy surgery took place in summer, the pinealectomy inhibited the regression of gonads that accompanied exposure of the animals to a shorter daylength [337]. If the pinealectomy surgery took place in winter, the testes accelerated in growth at a significantly faster rate once the garden lizards were exposure to a longer daylength [337]. These findings support a photoperiod-dependent role for seasonal, pineal-derived melatonin on the reproductive state of male lizards. However, the differences in lighting conditions for animals housed for these experiments should be noted. Interestingly, pinealectomies in parthenogenetic whiptails (*Cnemidophorus uniparens*) did not disrupt circannual reproductive cycles (vitellogenesis, ovulation, and oviposition) in 11 out of 13 females, but the pinealectomy itself appeared to cause high mortality with only 13 out of 32 animals surviving the surgery [338]. However, it is possible that this high rate of mortality was due to systematic error.

Among amphibia, Anura and Urodela are the foci of pineal and melatonin research to date [256]. Melatonin is involved in the neuroendocrine control of spawning in several anuran species [339]. In *Pelophylax perezi* (previously *Rana perezi*), circulating levels of melatonin are more closely linked to ocular production of melatonin than to pineal, but diurnal, rhythmic fluctuations of plasma melatonin concentrations are only observed at higher temperatures in summer and are abolished at lower temperature ranges [340]. Additionally, tissue sensitivity to melatonin in *P. perezi* appears to fluctuate diurnally in a light-dependent manner but varies with neither season nor temperature [341]. In female *P. perezi*, pinealectomy and partial blinding corresponded to significantly higher circulating oestradiol concentrations relative to sham-operated controls [342]. Furthermore, ovarian regression typically induced by high temperature ranges was prevented by pinealectomy and partial blinding [342]. However, these physiological consequences can not be solely ascribed to possible changes in melatonin, for changes in melatonin concentrations following pinealectomy were not determined in [342]. Because male *P. perezi* ocular melatonin production correlated with plasma melatonin concentrations, but melatonin concentrations within the pineal complex did not, it is reasonable to predict that partial blinding and pinealectomy in female *P. perezi* would fail to eradicate diurnal fluctuations in the melatonin production of this anuran. In male bullfrogs (*Rana catesbeiana*) raised in the lab, photoperiod did not significantly influence spermiation following LH/FSH injection, and temperature was a driving factor, with 30 °C preventing recrudescence and 15 °C/20 °C significantly reducing spermiation [343]. This might be attributed to the unnatural photoperiodic history of this lab-reared population. Female bullfrogs (*R. catesbeiana*), both wild-caught and lab-reared, housed in 12L:12D, but not longer (20L:4D) nor shorter (4L:20D) photoperiods, had significantly reduced atresia and prevented ovarian regression [344]. However, this study used unnaturally long and short photoperiods and does not serve us in understanding the influence of more subtle fluctuations of seasonal photoperiod. Ecologically relevant evidence in support of photoperiod as the primary cue informing anuran reproductive state can be supported by acoustic identification of anuran activity at Espinas stream in Maldonado, Uruguay over the course of two years [345]. Neither rainfall nor temperature significantly affected the richness of the identified calls, but photoperiod positively correlated with species richness based on acoustic identification [150]. While this might be due to only a subset of species calling during long days, hence the observed increase in richness during long days, this study elegantly captures a behavior associated with breeding activity (related to territoriality or mate solicitation) in a collective wild population. Given previous work reviewed here associating seasonal fluctuations in nocturnal melatonin with photoperiod, melatonin is a strong candidate for the chemical transducer of photic information and should be investigated in the subset of species that only called during long days.

In fishes, Teleostei is the largest group studied to date on melatonin and reproductive state, but studies in Chondrichthyes are limited to the role of melatonin in luminescent photophore patterns (in lantern shark, *Etmopterus spinax* [150], and in pygmy shark, *Squaliolus aliae* [346]) or in non-visual receptors (in the elephant shark, *Callorhinchus milii* [347]), which are outside the realm of melatonin’s involvement in the HPG axis reviewed here (for general review on HPG axis in elasmobranchs, see [348]). However, there are extensive reviews on the role of melatonin in teleost reproduction, including melatonin receptor distribution in the hypothalamus [349], the effects of photoperiod and temperature on reproduction [350,351,352], circannual rhythms and mate selection [353]. Some teleosts spawn in long days while others use decreasing daylength as a cue for spawning, and melatonin injections seems to have no significant effect on the reproductive state of the former and suppresses reproductive development in the latter [256]. Furthermore, continuous administration of melatonin in silastic capsules or in water of different species has inconsistent effects on variables related to reproductive development [256]. However, photoperiod plays an important role in seasonal reproduction in teleosts [256]. Given that salinity [354] and temperature [351] affect daily melatonin rhythms in some teleosts, we must consider how smolting and other fish-specific behaviors coincide with photoperiod. Although there is substantial evidence for hypothalamic binding of melatonin in teleosts [349], the field has limited studies testing melatonin receptor antagonists to distinguish the action of melatonin via its receptor from its role as an antioxidant (for review of the antioxidant role of melatonin in fish reproduction, see [355]). Furthermore, we have only begin to scratch the water’s surface to the effects of anthropogenic sources of light at night that, even with diffraction, is detectable by underwater species [356].

## 5. Conclusions

“When we turn from description to causal analysis, and ask in what way the observed change in behaviour machinery has been brought about, the natural first step to take is to try and distinguish between environmental influences and those within the animal. It is about this very first, preliminary step that confusion has arisen” [160].

The classical HPG axis and melatonin do not operate disparately. Interdependent variables are fundamentally changed when they are experimentally isolated to ascertain causality. Countless endogenous and plastic physiological factors, as well as the perception and transduction of predictable and unpredictable environmental cues, coordinate in a manner that has evolved over time to adapt to ever-changing environments. These endogenous or entrained physiological responses are not guaranteed to enhance survival and reproductive fitness across contexts. Reviews lab-field experiments [59], eco-endo-immunology [357], and the influences of stress response on breeding [358] offer a few examples of how experimental and environmental context affects the HPG axis. Inspired by Tinbergen’s four questions [160] and application for integrating research in GnIH [359], procedural questions for future studies in melatonin and reproduction are summarized in Table 2.

Regarding the questions related to phylogeny, it is important to note Tinbergen’s parameters on such studies in ethology:

“With the growing trend towards experimentation it is important, however, to point out that even the most perfect experiment of this kind does not give us direct proof of what selection has done in the past. The interpretation of such experiments as contributions to evolution theory will always include an extrapolation: while they demonstrate what selection can do, the best they can tell us is that selection can have happened in the way demonstrated, and that the results obtained are not contradictory to what other indirect evidence has led us to suppose. They really deal merely with “possible future evolution”, and only indirectly with past evolution” [160].

The variability in melatonin synthesizing and binding sites across species was reviewed here. Studies using pinealectomies that concluded melatonin has no effect on the reproductive state of a non-mammalian species only mention potential compensatory mechanisms of extra-pineal sources of melatonin to transduce photoperiodic information. Future studies should consider using gene editing methodologies to locally downregulate or upregulate melatonin-synthesizing enzymes and/or melatonin receptors to ascertain the effects on reproductive nucleotide transcription, translation, and associated changes in reproductive physiology.

There is also evidence in support of the role of melatonin in driving reproductive state in photoperiodic, seasonally breeding mammals. The mechanism underlying melatonergic regulation of mammalian gonadal steroid production might be conserved because melatonin appears to have a similar effects in non-mammalian vertebrates as well (in European starling testes [133], in *Bufo arenarum* oocytes [360], in teleost testes, *Fundulus similis*, and in tree frog testes, *Hyla cinerea* [361]).

To conclude, physiological effects of melatonin are observed at all levels of the classical HPG axis in seasonally breeding species classified from different vertebrate classes. Melatonin can influence these effects through its role as an antioxidant or by binding and activating its specific subtype receptors. The para- and autocrine signalling taking place in the brain has far more extensive research than what has been studied in the pituitary and the gonads. The presence of GnRH and GnIH receptors in the gonads, as well as local synthesis of these “neuropeptides” [179,362], opens a whole new field for research in the effects of melatonin on gonadal neuropeptides and how these effects compare to effects on neuropeptides in the brain. The potential for melatonergic mechanisms to alter ontologically, provide adaptive value, and share conserved characteristics that are comparative in nature stimulates innumerable inquiries for seasonal reproductive endocrinology.

## Figures and Tables

**Table 1:** NCBI accession numbers and full names of sequences used for phylogenetic analysis. All sequences that were available for the selected primates, rodents, and non-mammalian species were used. Species line up horizontally on this table. Mel1C has not been reported in mammals. NQO2/QR2 has been reported to be the putative MT3 receptor. Sequences were curated using Clustal Omega Multiple Sequence Alignment.

Mel1a/MTNR1A		Mel1b/MTNR1B		Mel1C/MTNR1C		NAD(P)H Dehydrogenase Quinone 2/NQO2 (Ribosyldihydronicotinamide Dehydrogenase)	
***MTNR1A_Homo sapiens***	NM_005958.4 Homo sapiens melatonin receptor 1A (MTNR1A), mRNA	***MTNR1B_Homo sapiens***	NM_005959.3 Homo sapiens melatonin receptor 1B (MTNR1B), mRNA			***NQO2_Homo sapiens***	J02888.1 Human quinone oxidoreductase (NQO2) mRNA, complete cds
***MTNR1A_Gorilla gorilla gorilla***	XM_004040725.2 PREDICTED: Gorilla gorilla gorilla melatonin receptor 1A (MTNR1A), mRNA	***MTNR1B_Gorilla gorilla gorilla***	XM_004051965.2 PREDICTED: Gorilla gorilla gorilla melatonin receptor 1B (MTNR1B), mRNA			***NQO2_Gorilla gorilla gorilla***	XM_019029756.1 PREDICTED: Gorilla gorilla gorilla NAD(P)H quinone dehydrogenase 2 (NQO2), transcript variant X8, mRNA
***MTNR1A_Macaca mulatta***	XM_001090972.3 PREDICTED: Macaca mulatta melatonin receptor 1A (MTNR1A), mRNA	***MTNR1B_Macaca mulatta***	XM_001084265.3 PREDICTED: Macaca mulatta melatonin receptor 1B (MTNR1B), mRNA			***NQO2_Macaca mulatta***	XM_015135430.1 PREDICTED: Macaca mulatta NAD(P)H dehydrogenase, quinone 2 (NQO2), transcript variant X3, mRNA
***MTNR1A_Marmota marmota marmota***	XM_015486060.1 PREDICTED: Marmota marmota marmota melatonin receptor 1A (Mtnr1a), mRNA	***MTNR1B_Marmota marmota marmota***	XM_015489993.1 PREDICTED: Marmota marmota marmota melatonin receptor 1B (Mtnr1b), mRNA			***NQO2_Marmota marmota marmota***	XM_015507543.1 PREDICTED: Marmota marmota marmota NAD(P)H dehydrogenase, quinone 2 (Nqo2), transcript variant X2, mRNA
***MTNR1A_Heterocephalus glaber***	XM_004853058.2 PREDICTED: Heterocephalus glaber melatonin receptor 1A (Mtnr1a), mRNA	***MTNR1B_Heterocephalus glaber***	XM_004838123.1 PREDICTED: Heterocephalus glaber melatonin receptor 1B (Mtnr1b), mRNA			***NQO2_Heterocephalus glaber***	XM_013073256.1 PREDICTED: Heterocephalus glaber NAD(P)H dehydrogenase, quinone 2 (Nqo2), transcript variant X2, mRNA
***MTNR1A_Peromyscus maniculatus***	XM_006970866.1 PREDICTED: Peromyscus maniculatus bairdii melatonin receptor 1A (Mtnr1a), mRNA	***MTNR1B_Peromyscus maniculatus bairdii***	XM_006990545.1 PREDICTED: Peromyscus maniculatus bairdii melatonin receptor 1B (Mtnr1b), mRNA			***NQO2_Peromyscus maniculatus bairdii***	XM_006983508.2 PREDICTED: Peromyscus maniculatus bairdii NAD(P)H dehydrogenase, quinone 2 (Nqo2), mRNA
***Mel1a_Phodopus sungorus***	U14110.1 Phodopus sungorus melatonin receptor Mel-1a mRNA, complete cds	***Mel1b_Phodopus sungorus***	U57555.1 Phodopus sungorus Mel1b melatonin receptor mRNA, partial cds				
***MTNR1A_Mus musculus***	NM_008639.2 Mus musculus melatonin receptor 1A (Mtnr1a), mRNA	***MTNT1B_Mus musculus***	NM_145712.2 Mus musculus melatonin receptor 1B (Mtnr1b), mRNA			***NAD(P)Hdehydrogenase_ quinone2_Mus musculus***	BC027629.1 Mus musculus NAD(P)H dehydrogenase, quinone 2, mRNA (cDNA clone MGC:41088 IMAGE:1225122), complete cds
***MTNR1A_Cricetulus griseus***	XM_007617174.2 PREDICTED: Cricetulus griseus melatonin receptor 1A (Mtnr1a), mRNA	***Mel1B_Cricetulus griseus***	XM_007636225.1 PREDICTED: Cricetulus griseus melatonin receptor type 1B-like (LOC103163272), mRNA			***NQO2_Cricetulus griseus***	XM_007638944.2 PREDICTED: Cricetulus griseus NAD(P)H quinone dehydrogenase 2 (Nqo2), transcript variant X2, mRNA
***Mel1a_Mesocricetus auratus***	AF061158.1 Mesocricetus auratus melatonin receptor Mel1a mRNA, partial cds	***Mel1b_Mesocricetus auratus***	AY145849 Mesocricetus auratus Mel1b melatonin receptor pseudogene, partial sequence			***NQO2_Mesocricetus auratus***	XM_013110593.1 PREDICTED: Mesocricetus auratus NAD(P)H dehydrogenase, quinone 2 (Nqo2), transcript variant X1, mRNA
***MTNR1A_Rattus norvegicus***	NM_053676.2 Rattus norvegicus melatonin receptor 1A (Mtnr1a), mRNA	***MTNR1B_Rattus norvegicus***	NM_001100641 Rattus norvegicus melatonin receptor 1B (Mtnr1b), mRNA			***NAD(P)Hdehydrogenase_ quinone2_Rattus norvegicus***	BC079157.1 Rattus norvegicus NAD(P)H dehydrogenase, quinone 2, mRNA (cDNA clone MGC:94180 IMAGE:7128990), complete cds
***MTNR1A_Gallus gallus***	NM_205362.1 Gallus gallus melatonin receptor 1A (MTNR1A), mRNA	***MTNR1B_Gallus gallus***	NM_001293103.1 Gallus gallus melatonin receptor 1B (MTNR1B), mRNA			***NQO2_Gallusgallus***	HAEL01011471.1 TSA: Gallus Gallus, breed ISA Brown, contig c17404/1/1559, transcribed RNA sequence
***Mel1A_Coturnix japonica***	XM_015862146.1 PREDICTED: Coturnix japonica melatonin receptor type 1A (LOC107313685), mRNA	***MTNR1B_Coturnix japonica***	XM_015852347.1 PREDICTED: Coturnix japonica melatonin receptor 1B (MTNR1B), transcript variant X1, mRNA			***NQO2_Coturnix japonica***	XM_015854496.1 PREDICTED: Coturnix japonica NAD(P)H dehydrogenase, quinone 2 (NQO2), mRNA
***MTNR1A_Meleagris gallopavo***	XM_003205776.3 PREDICTED: Meleagris gallopavo melatonin receptor 1A (MTNR1A), mRNA	***Mel1B_Meleagris gallopavo***	XM_010706129.2 PREDICTED: Meleagris gallopavo melatonin receptor type 1B (LOC104909381), mRNA			***NQO2_Meleagris gallopavo***	XM_010708413.2 PREDICTED: Meleagris gallopavo NAD(P)H quinone dehydrogenase 2 (NQO2), mRNA
***MTNR1A_Sturnus vulgaris***	XM_014873402.1 PREDICTED: Sturnus vulgaris melatonin receptor 1A (MTNR1A), mRNA	***MTNR1B_Sturnus vulgaris***	XM_014877499.1 PREDICTED: Sturnus vulgaris melatonin receptor 1B (MTNR1B), mRNA			***NQO2_Sturnus vulgaris***	XM_014872375.1 PREDICTED: Sturnus vulgaris NAD(P)H dehydrogenase, quinone 2 (NQO2), mRNA
***MTNR1A_Taeniopygia guttata***	NM_001048257.1 Taeniopygia guttata melatonin receptor 1A (MTNR1A), mRNA	***MTNR1B_Taeniopygia guttata***	NM_001048258.1 Taeniopygia guttata melatonin receptor 1B (MTNR1B), mRNA			***NAD(P)Hdehydrogenase_ quinone2_Taeniopygia guttata***	DQ216354.1 Taeniopygia guttata clone 0065P0009D12 putative NAD(P)H dehydrogenase quinone 2 variant 2 mRNA, complete cds
***MTNR1A_Columba livia***	XM_005500227 PREDICTED: Columba livia melatonin receptor 1A (MTNR1A), mRNA	***Mel1B_Columba livia***	XM_005512954.2 PREDICTED: Columba livia melatonin receptor type 1B (LOC102095424), mRNA				
***MTNR1A_Parus major***	XM_019006224 PREDICTED: Parus major melatonin receptor 1A (MTNR1A), mRNA	***MTNR1B_Parus major***	XM_015647149 PREDICTED: Parus major melatonin receptor 1B (MTNR1B), mRNA			***NQO2_Parus major***	XM_015616869.1 PREDICTED: Parus major NAD(P)H quinone dehydrogenase 2 (NQO2), partial mRNA
***MTNR1A_Alligator sinensis***	XM_014521485 PREDICTED: Alligator sinensis melatonin receptor 1A (MTNR1A), mRNA	***Mel1B_Alligator sinensis***	XM_006021055.1 PREDICTED: Alligator sinensis melatonin receptor 1B (MTNR1B), partial mRNA			***NQO2_Alligator sinensis***	XM_014519268.1 PREDICTED: Alligator sinensis NAD(P)H dehydrogenase, quinone 2 (NQO2), transcript variant X1, mRNA
		
***MTNR1A_Alligator mississippiensis***	XM_006262998 PREDICTED: Alligator mississippiensis melatonin receptor 1A (MTNR1A), transcript variant X1, mRNA	***Mel1B_Alligator mississippiensis***	XM_006276749.3 PREDICTED: Alligator mississippiensis melatonin receptor type 1B (LOC102567490), partial mRNA			***NQO2_Alligator mississippiensis***	XM_019490389.1 PREDICTED: Alligator mississippiensis NAD(P)H quinone dehydrogenase 2 (NQO2), transcript variant X2, mRNA
		
***MTNR1A_Chelonia mydas***	XM_007062677 PREDICTED: Chelonia mydas melatonin receptor type 1A-like (LOC102946412), mRNA	***Mel1B_Chelonia mydas***	XM_007072335.1 PREDICTED: Chelonia mydas melatonin receptor type 1B-like (LOC102947942), mRNA				
***MTNR1A_Pelodiscus sinensis***	XM_006128505 PREDICTED: Pelodiscus sinensis melatonin receptor 1A (MTNR1A), mRNA	***Mel1B_Pelodiscus sinensis***	XM_014577564.1 PREDICTED: Pelodiscus sinensis melatonin receptor type 1B (LOC102444642), mRNA			***NQO2_Pelodiscus sinensis***	XM_014581445.1 PREDICTED: Pelodiscus sinensis NAD(P)H dehydrogenase, quinone 2 (NQO2), mRNA
***Mel1A_Xenopus laevis***	XM_018243177 PREDICTED: Xenopus laevis melatonin receptor type 1A (LOC108706613), mRNA	***Mel1B_Xenopus laevis***	XM_018250356 PREDICTED: Xenopus laevis melatonin receptor type 1B (LOC108710014), mRNA	***Mel1Ca_Xenopus laevis***	U67880 Xenopus laevis Mel-1c(a) melatonin receptor long mRNA, complete cds		
				***Mel1Cb_Xenopus laevis***	U67882 Xenopus laevis Mel-1c(b) melatonin receptor long mRNA, complete cds		
				***MTNR1C_Xenopus laevis***	KF486518 Xenopus laevis melatonin receptor type 1C (MTNR1C) mRNA, complete cds, alternatively spliced		
***MTNR1A_Xenopus tropicalis***	XM_002940864.3 PREDICTED: Xenopus (Silurana) tropicalis melatonin receptor 1A (mtnr1a), mRNA [removed from NCBI]	***MTNR1B_Xenopus tropicalis***	XM_004920744 PREDICTED: Xenopus tropicalis melatonin receptor 1B (mtnr1b), partial mRNA	***MTNR1C_Xenopus tropicalis***	XM_004916882 PREDICTED: Xenopus tropicalis melatonin receptor type 1C (mtnr1c), mRNA		
***MTNR1A_Nanorana parkeri***	XM_018563328 PREDICTED: Nanorana parkeri melatonin receptor 1A (MTNR1A), mRNA	***Mel1B_Nanorana parkeri***	XM_018558631 PREDICTED: Nanorana parkeri melatonin receptor type 1B (LOC108788772), mRNA	***Mel1C_Nanorana parkeri***	XM_018552598 PREDICTED: Nanorana parkeri melatonin receptor type 1C (LOC108783900), mRNA		
***Mtnr1Aa_Danio rerio***	NM_131393 Danio rerio melatonin receptor 1A a (mtnr1aa), mRNA	***Mel1Ba_Danio rerio***	BC163408 Danio rerio melatonin receptor type 1B a, mRNA (cDNA clone MGC:194859 IMAGE:9038309), complete cds	***MTNR1C_Danio rerio***	NM_001161484 Danio rerio melatonin receptor 1C (mtnr1c), mRNA		
***Mtnr1Ab_Danio rerio***	XM_688989 PREDICTED: Danio rerio melatonin receptor 1A b (mtnr1ab), mRNA						
***MTNR1A_Pygocentrus nattereri***	XM_017704098 PREDICTED: Pygocentrus nattereri melatonin receptor 1A (mtnr1a), transcript variant X1, mRNA	***Mel1B_Pygocentrus nattereri***	XM_017691296.1 PREDICTED: Pygocentrus nattereri melatonin receptor type 1B-B (LOC108423763), mRNA	***Mel1Clike_Pygocentrus nattereri***	XM_017713350 PREDICTED: Pygocentrus nattereri melatonin receptor type 1C-like (LOC108436701), mRNA	***RibosyldihydronicotinamideDehydrogenase_Pygocentrus nattereri***	XM_017684980.1 PREDICTED: Pygocentrus nattereri ribosyldihydronicotinamide dehydrogenase [quinone]-like (LOC108412776), mRNA
				***Mel1C_Pygocentrus nattereri***	XM_017721904 PREDICTED: Pygocentrus nattereri melatonin receptor type 1C-like (LOC108442058), mRNA		
***Mel1Alike_Latimeria chalumnae***	XM_014496132 PREDICTED: Latimeria chalumnae melatonin receptor type 1A-like (LOC102351371), partial mRNA	***Mel1B_Latimeria chalumnae***	XM_014492552 PREDICTED: Latimeria chalumnae melatonin receptor type 1B (LOC102361192), transcript variant X2, mRNA				

**Table 2:** Tinbergen’s four questions applied to research in melatonin and seasonal reproduction.

**Proximate**
*Mechanism* Has a circadian rhythm for melatonin synthesis/secretion been determined in this species as is being used for the experiment (e.g., age, sex, photoperiodic history, lighting schedule, wild-caught in the same region vs. laboratory raised, etc.), enabling melatonin to serve as a chemical transducer of photoperiodic information in this species? Are there daily or annual fluctuations in concentrations of melatonin within specific tissues or circulating in plasma? How does melatonin receptor affinity and density vary within the brain and/or gonads of this species at different times of the day/year? *Ontogeny* How does melatonin synthesis/secretion change with development? At what stage of development does the species become fertile? How does the onset of puberty compare to seasonal gonadal development or recrudescence? How does senescence compare to seasonal gonadal regression? (for review, see Perfito & Bentley, 2009)
**Ultimate**
*Adaptive Value* Is there a selective advantage to using photoperiod as a cue for reproduction in this species, given their geographical place of origin or current distribution in the wild? Has this species been selectively bred in an environment that is comparable to where they adapted/evolved in the wild? How many seasons is this species fertile? Would using photoperiod over multiple seasons provide an advantage for survival or resource acquisition in the environment where they breed? *Phylogeny* Are there species of other vertebrate classes that can be used for comparison? What examples for homology or convergence can be considered for understanding melatonin and reproductive timing in this species? Can controlled selective pressure to become a photoperiodic breeder or lose photoperiodic responsiveness change how melatonin affects the reproductive axis over multiple generations?
